# Die Meldepflicht als Grundlage der epidemiologischen Statistik: Die Auswirkungen der Meldepraxis und der Verwendung von *paper technologies* auf den Informationsgehalt von Morbiditätsstatistiken 1886–1921

**DOI:** 10.1007/s00048-024-00375-4

**Published:** 2024-02-06

**Authors:** Henrik Jochum

**Affiliations:** Institut für Biomedizinische Ethik und Medizingeschichte (IBME), Universität Zürich, Zürich, Schweiz

**Keywords:** Meldepflicht, Meldewesen, Historische Statistiken, *Paper technologies*, Practical turn, Mandatory reporting, Reporting systems, Historical statistics, *Paper technologies*, Practical turn

## Abstract

Dieser Artikel untersucht anhand einer Betrachtung der praktischen Umsetzung des schweizerischen Meldewesens für Infektionskrankheiten zwischen 1886 und 1921, welche Auswirkungen die Meldepraxis und die Verwendung von *paper technologies *auf die Meldungen hatten, die daraufhin für Morbiditätsstatistiken verwendet wurden. Eine genaue Untersuchung der Herstellungsprozesse von Meldungen zeigt die Schwierigkeiten und Lösungsansätze bei der Umsetzung des gesetzlich vorgegebenen Meldeprozesses. Zwei Krankheitsausbrüche – ein Pockenausbruch in Schaffhausen und ein Typhusausbruch im Kanton Luzern – dienen dabei als Fallbeispiele. Es wird gezeigt, dass Meldungen mehr als nur objektive Repräsentationen von Erkrankungen sind, sondern auch die medizinisch-sozialen Interaktionen darstellen, die sie produzieren, gezeitigt durch administrative Werkzeuge wie Meldeformulare und den Akt des Meldens. Das problematisiert die Aussagekraft von historischen Statistiken und zeigt die Komplexität des historischen Quellenmaterials, da diese Interaktionen und ihre Auswirkungen auf die Meldungen mit in Betracht gezogen werden müssen. Diese Erkenntnisse werden mit der Betrachtung des schweizerischen Meldewesens während der Spanischen Grippe 1918 und dessen Scheitern bei der Erfassung der Influenzafälle verdeutlicht.

## Abstract (en)

This article examines the impact of the reporting practice and *paper technologies *like forms on reports that were later used for national morbidity statistics by studying the Swiss reporting system for infectious diseases between 1886 and 1921. Analysing the production processes of notifications shows the difficulties and solutions in the implementation of the statutory reporting process. Two disease outbreaks—a smallpox outbreak in Schaffhausen and a typhoid outbreak in the canton of Lucerne—serve as case studies. It is shown that reports are not only objective representations of diseases, but also symbolize the medico-social interactions that produce them, timed by administrative tools like reporting forms and the act of reporting. This destabilises historical statistics and illustrates the complexity of the historical source material, as these interactions and their impact on reporting must be considered. These findings are further supported by examining the Swiss reporting system during the Spanish flu of 1918 and its failure to record influenza cases.

Unleserliche ärztliche Handschriften entziffern, stapelweise Meldeformulare auf eine Waage legen, um sie zu wiegen statt zu zählen, um damit irgendwie zeitnah eine ungefähre Fallzahl kommunizieren zu können, auf Faxe warten. So musste das Schweizerische Bundesamt für Gesundheit im März 2020 versuchen, die gemeldeten Fälle von COVID-19 zu erfassen (Fichter 2020). Die Herausforderung des immensen Volumens an Meldungen wurde erschwert durch eine veraltete IT-Struktur, komplizierte Meldeprozesse und unverbindliche Meldekriterien. Bei einem Großteil der Mitteilungen fehlten wichtige Zusatzinformationen, wie etwa wo und wann die Ansteckung erfolgt oder ob die erkrankte Person wieder genesen war. Teilweise nutzten Mitarbeitende beim Aufstellen der offiziellen Statistik die von der Presse oder anderen Kanälen wie Wikipedia angegebenen Zahlen, die sie von Medienmitteilungen und kantonalen Lagebulletins übernahmen, und erst später das eigene Meldesystem.

Die COVID-19-Pandemie hat gezeigt, welche zentralen Rollen Fallzahlen und Statistiken sowie daraus erschlossene epidemiologische Aussagen bei der Bekämpfung von ansteckenden Krankheiten spielen. Politische Entscheidungen wie das Ergreifen und Lockern von Massnahmen waren teils an spezifische Zahlenwerte wie die Reproduktionszahl – bei deren Berechnung die gemeldeten Fälle mehrerer Tage verwendet werden – oder die 7‑Tage-Inzidenz – dem Verhältnis der Anzahl Fälle zur jeweiligen Einwohnerzahl – gebunden (Bundesamt für Gesundheit 2022). Doch wie die obige Episode aus dem Meldewesen der Schweiz illustriert, sind die Herstellungsprozesse dieser Daten mit diversen Schwierigkeiten verbunden. Das verwundert, da die Schweiz bereits seit 1886 ein bundesweites Epidemiengesetz und ein entsprechendes Meldewesen für ansteckende Krankheiten hat. Dieses seit über 130 Jahren bestehende System der Epidemienbekämpfung und -erfassung – das unter anderem bereits mit Herausforderungen wie der Spanischen Grippe konfrontiert war – hatte im Frühjahr 2020 Schwierigkeiten, adäquat auf den Ausbruch von COVID-19 zu reagieren. Die darauffolgenden Verbesserungen im schweizerischen Meldewesen in den Folgemonaten zeigten es als historischen Gegenstand und verlangen eine nähere Betrachtung der Geschichte der Meldepflicht sowie ihrer praktischen Umsetzung.

Im deutschsprachigen Raum haben sich bisher einige geschichtswissenschaftliche Untersuchungen mit der normativen Grundlage der Meldepflicht – den Gesetzgebungen zur Seuchenbekämpfung – beschäftigt (Ruckstuhl & Ryter 2017; Hess 2009; Hüntelmann 2008). In England hat Graham Mooney das dortige Meldewesen für ansteckende Krankheiten betrachtet und untersucht, wie durch die zunehmende staatliche Überwachung der Epidemienprävention und -bekämpfung Ende des 19. Jahrhunderts Staatsbürgerschaft neu definiert wurde (Mooney 2015). Auch in Anne Hardys Betrachtung des Aufkommens der Präventivmedizin in Großbritannien zu dieser Zeit spielt die Meldepflicht eine wichtige Rolle als Werkzeug für die Erfassung von ansteckenden Krankheiten (Hardy 1993). Im Kontext von Tuberkulose wurden die Konsequenzen einer Meldung für die Betroffenen beschrieben sowie angedeutet, dass die Diagnose von Infektionskrankheiten in der Praxis zu Beginn des 20. Jahrhunderts – trotz vorhandener laboratorischer Tests – noch immer eine große Herausforderung darstellte und entsprechend von den meldenden Individuen abhängig war (Bryder 1988). Auf diesen Arbeiten aufbauend ergänzt der vorliegende Beitrag eine nähere Untersuchung der schweizerischen Meldepflicht und Meldepraxis im Kontext von für Morbiditätsstatistiken ansteckender Krankheiten verwendeten Meldungen.

Die Meldepflicht stellt dabei ein essenzielles Zahnrad in der seuchenpolizeilichen Infrastruktur sowie der Erstellung von Medizinalstatistiken und damit auch in der Erfassung von Infektionskrankheiten dar. Gerade die Erstellung von Statistiken zu Krankheitsfällen war während des Aufbaus der modernen Nationalstaaten Ende des 19. Jahrhunderts und der Etablierung moderner Gesundheitspolitiken eine administrative Herausforderung (Hüntelmann 2019: 43; Thiessen 2014: 17f.). Diese Statistiken, die eine ungefähre Bezifferung der Betroffenen erlauben, sind vielgenutzte Primärquellen in der Seuchengeschichte, um den Umfang und Verlauf von vergangenen Epidemien abzubilden (Kavey & Kavey 2021; Vögele et al. 2016; Vasold 2008; Winkle 2005; Leven 1997). Das ist verständlich, da sie einen Reichtum an Informationen enthalten, der bei früheren Epidemien wie etwa der großen Pest in Europa zwischen 1347 und 1353 grösstenteils fehlt (Belich 2022; Reinhardt 2021). Auch werden sie genutzt, um vergangene Epidemien wie die Spanische Grippe und bekämpfende Massnahmen mithilfe gegenwärtiger epidemiologischer Methoden zu untersuchen (Staub et al. 2021). Die Limitationen und die Historizität von Medizinalstatistiken sowie medizinischen Daten wurden dabei in der Medizin- und Wissenschaftsgeschichte viel und kritisch diskutiert (Janssens & Devos 2022; Hüntelmann & Falk 2021; Haas et al. 2019; Jorland & Weisz 2005).

Eine Perspektive, der hier mehr Aufmerksamkeit gebührt und die wichtige Erkenntnisse für die Verwendung der Statistiken als geschichtswissenschaftliche Quellen erlaubt, ist die praktische Umsetzung der Meldepflicht. Die Anthropologin Annemarie Mol hat mit *The Body Multiple *die Vielfalt aufgezeigt, in der die gleichen medizinische Größen in der Praxis verstanden werden und wie sich Handlungsanweisungen sowie ihre praktische Umsetzung unterscheiden (Mol 2002). Dies dient als methodische Anregung und Inspiration, die Meldepraxis aus einer sozial- und medizinhistorischen Perspektive näher zu hinterfragen. Wie dieser Beitrag zeigen wird, sind die Bedeutungen der erfassten und statistisch wiedergegebenen Pocken‑, Typhus- und Grippefälle teils weit entfernt von ihrem medizinischen Ursprung. Das Ziel der Untersuchung ist dabei, durch eine Betrachtung der Meldepraxis und damit der Produktionsprozesse von Meldungen aufzuzeigen, wie sich die Herausforderungen der Umsetzung der Meldepflicht auf deren Inhalt auswirkten. Eine solche Betrachtung soll auch aus einer methodischen Perspektive zur Herangehensweise und kritischen Reflexion von historischen, aber auch gegenwärtigen Statistiken bei deren Verwendung beitragen. Denn genau diese praktische Umsetzung der Meldepflicht war es auch, die im eingangs beschriebenen Beispiel der Erfassung von COVID-19 im März 2020 verwunderte und eine eingehende Reflexion auslöste. Der Beitrag liegt dabei an der Schnittstelle zwischen der Geschichte der medizinischen Statistik, administrativer Organe wie der Meldepflicht, Arbeitsdokumenten wie Formularen und dem *practical turn*. Ich untersuche dabei, was die Aufgaben des Meldewesens für Infektionskrankheiten in der Schweiz ab 1886 waren, wie es umgesetzt wurde und was passierte, sobald es mit einer großflächigen Epidemie – der Spanischen Grippe ab 1918 – konfrontiert war. Zentrale Themen in der Studie sind dabei die normativen Vorgaben der statistischen Erfassung von Infektionskrankheiten in der Schweiz sowie ihre praktische Umsetzung, die Herstellungsprozesse der Meldungen, die Diagnose und Feststellung von Infektionskrankheiten um 1900 sowie was sie beeinflusste und der Einfluss von administrativen Werkzeugen wie Meldeformularen auf den Meldeprozess und den Informationsgehalt von Statistiken zu ansteckenden Krankheiten.

Obwohl es nicht möglich sein wird, im gleichen Detail wie Annemarie Mols Studie die medizinische Praxis zu untersuchen, wird trotzdem versucht, sie so genau wie möglich zu erfassen. Diese Nähe soll unter anderem mithilfe von in der Meldepraxis verwendeten *paper technologies *– im nachfolgenden Beitrag sind dies Meldeformulare – aufgebaut werden. Die Formulare tauchen hier als eine zentrale Quelle und Verknüpfung zwischen medizinischer Praxis sowie staatlicher Statistik auf. Dabei haben sie eine Doppelrolle sowohl als geschichtswissenschaftliche Quelle als auch als Quelle für die medizinischen Statistiken. Ähnlich wie bereits Christine von Oertzen im Sammelband *Working with Paper* beschrieben hat, waren Formulare als *paper technologies* essenzielle staatliche Arbeitsdokumente, um statistische Erfassungen zu vereinfachen und zu beschleunigen (von Oertzen 2019: 108). Gerade auch in der medizinischen Praxis spielten diverse Verschriftlichungsformen essenzielle Rollen bei der Herstellung und Erfassung von Wissen, wie der Sammelband *Accounting for Health* gezeigt hat (Hüntelmann & Falk 2021). Die nachfolgende Untersuchung nimmt dabei eine ähnliche Perspektive wie diese beiden Studien ein und interessiert sich für die praktische Umsetzung der Meldepflicht durch Formulare und wie sie sich auf das von ihnen produzierte Wissen, das daraufhin in den medizinischen Statistiken verarbeitet wurde, ausgewirkt hat. Dabei steht ihre Rolle im administrativen Arbeitsprozess im Vordergrund und weniger, welchen Einfluss die Materialität und Schriftlichkeit der Dokumente hatte.

Dieser Beitrag konzentriert sich dabei auf die Herstellung von Meldungen, die daraufhin in die schweizerische Morbiditätsstatistik einflossen. Obwohl gewisse Schwierigkeiten wie etwa die Diagnosestellung bei beiderlei Morbiditäts- und Mortalitätsstatistiken auftreten, unterscheiden sie sich wesentlich in Aspekten wie etwa dem zeitlichen Verlauf einer Krankheit und sich dadurch womöglich ändernden Diagnosen oder den Konsequenzen einer fehlenden Meldung für die Epidemienbekämpfung. Mortalitätsstatistiken und die Herausforderungen ihrer Verwendung als historische Quellen wurden dabei in einer Sonderausgabe von *Social Medicine *näher betrachtet (Janssens & Devos 2022).

In dieser Untersuchung versuche ich, die historischen Diagnosen als Denkprozesse der Akteur:innen bei der Herstellung der Meldungen von Infektionskrankheiten nachzuvollziehen. Im Sinne von Andrew Cunningham interessiere ich mich hierbei dafür, „how diagnosis happens“ (Cunningham 2002: 16). Weniger relevant ist die Feststellung der Krankheitsfälle im Sinne einer retrospektiven Diagnose, da es die zeitgenössischen Interpretationen in den Meldungen waren, die in die Statistiken einflossen und die ich hier näher betrachten möchte (Leven 1998). Gerade für das 20. Jahrhunderts bieten diese Statistiken einen reichhaltigen und wertvollen Einblick in die Vergangenheit, weshalb eine umfangreiche Kontextualisierung und Historisierung derselben zentral ist.

Das schweizerische Meldewesen ist in diesem Zusammenhang ein interessanter Untersuchungsgegenstand, da die Schweiz bereits während des betrachteten Zeitraums ein kleinräumiger Bundesstaat bestehend aus 25 teilsouveränen Kantonen war, der sich im europäischen Vergleich durch seine direkte Demokratie und den daraus resultierenden Föderalismus auszeichnete. Das bedeutete auch für die Epidemienbekämpfung, dass Lösungen in kleinen Organisationseinheiten gesucht und umgesetzt wurden. Diese Organisationseinheiten sind in der Struktur der Archivquellen wiedergegeben und erlauben dadurch eine detaillierte Betrachtung der Meldepraxis. Zudem war die Aufteilung der Kompetenzen zwischen dem Bund und den einzelnen Kantonen in der Schweiz ein fortwährendes Spannungsfeld, das konstant verhandelt wurde und worauf auch die Bevölkerung durch Initiativen sowie Referenden direkten Einfluss nehmen konnte. Das führte dazu, dass bei der Umsetzung der Meldepflicht aufgrund der umfangreichen Kompetenzen der Kantone diverse regionale Unterschiede bestanden. Diese Heterogenität und entsprechende Vielzahl an Akteuren wirkte sich auf die Meldepraxis und somit auch auf die historischen Statistiken aus, was aufgrund der Kleinräumigkeit der Systeme eine Untersuchung der Schnittstelle von normativen Vorgaben, praktischer Umsetzung und statistischen Informationen, für die ich mich interessiere, begünstigt.

Der erste Teil meiner Untersuchung beschäftigt sich daher mit der normativen Grundlage des schweizerischen Meldewesens – dem Epidemiengesetz von 1886 – und den Zielsetzungen der Meldepflicht. Obwohl das Gesetz und seine Vorgeschichte bereits Thema einiger geschichtswissenschaftlicher Untersuchungen war, muss für die nachfolgende Untersuchung die spezifische normative Grundlage der Meldepflicht nochmals genauer betrachtet werden (Yersin 2022; Ruckstuhl & Ryter 2017). Damit wird aufzeigt, für welche Situationen das Meldesystem in der Schweiz ausgelegt war und wie es funktionieren sollte. Es zeigt sich dabei als eine retrospektive Gesetzgebung, die sich nur auf vier immer seltener werdende Krankheiten bezog. Da die Umsetzung bei den Kantonen lag, untersuche ich daraufhin im zweiten Abschnitt die alltägliche Meldepraxis mithilfe zweier Fallbeispiele kleinerer Krankheitsausbrüche. Die Umsetzung basierte dabei bei den bundesstaatlich meldepflichtigen Krankheiten auf den kantonal individuellen Vollziehungsverordnungen, welche die jeweilige Auslegung des Epidemiengesetzes regelten, sowie auf den kantonalen Seuchengesetzen, die sich beide gegenseitig ergänzten. Aus diesem Grund wurden zwei verschiedene Kantone und zwei unterschiedliche Krankheiten als Beispiele gewählt, um die praktische Breite des schweizerischen Meldewesens und die entsprechenden Auswirkungen auf die Statistiken zu illustrieren. Die Betrachtung der Umsetzung erfolgt anhand eines Pockenausbruchs in der Stadt Schaffhausen 1903 und eines Typhusausbruchs in einem ländlichen Gebiet des Kantons Luzern 1904. Im Archiv erhaltene Berichte von Ärzten und administrative Dokumente wie Meldeformulare erlauben hier einen Einblick in die praktische Umsetzung der Meldeprozesse.^1^ Im dritten Teil wird die Meldepflicht und das Epidemiengesetz mit ihrer ersten großen Zerreißprobe konfrontiert: der Spanischen Grippe ab 1918. Hier zeigt sich, wie die administrativen Praktiken der Meldepflicht auf eine Epidemiesituation reagierten, auf deren Umfang und Ausgangslage sie nicht vorbereitet waren, und wie sich das auf die produzierten Informationen auswirkte.

## Das Epidemiengesetz von 1886 und die Meldepflicht der Schweiz

Gesundheit ist Kantonssache. Etwas überspitzt formuliert, beschreibt das seit der Gründung des modernen Schweizer Bundesstaats im Jahr 1848 die Aufteilung der diesbezüglichen Kompetenzen zwischen Staat und Kanton. Entsprechend lagen auch die Prävention und Bekämpfung von ansteckenden Krankheiten in erster Linie bei den Kantonen. Wie kam es dann 1886 zur Einführung eines bundesweiten Epidemiengesetzes sowie einhergehend damit zu einer staatlichen Meldepflicht, und wie wurde sie gestaltet? Die Bundesverfassung von 1848 gewährte dem schweizerischen Staat nur beim Auftreten von bestimmten Seuchen, reaktiv gesundheitspolizeiliche Verfügungen zu erlassen (Bundesverfassung 1848). Erst mit der Verfassungsrevision von 1874 und dem damit eingeführten Artikel 69 war er berechtigt, eine entsprechende Gesetzgebung und damit präventive Massnahmen einzuführen sowie auch ein bundesweites Meldewesen anzustreben (Bundesverfassung 1874). Das war Teil des Auf- und Ausbaus staatlicher Verwaltungen in der Schweiz aufgrund der im Verlauf des 19. Jahrhunderts aufkommenden gesundheitspolitischen Aufgaben (Ruckstuhl & Ryter 2017: 9). Die bundesweite Regelung von Seuchenmaßnahmen war bereits in den 1860er Jahren in Anbetracht von drohenden Pocken- und Choleraepidemien seitens einzelner Kantone angeregt worden (117). Ausgehend davon und basierend auf der verfassungsrechtlichen Grundlage von 1874 begann 1879 die dafür eingesetzte eidgenössische Sanitätskommission bestehend aus medizinischen Experten wie Jakob Laurenz Sonderegger – zu dieser Zeit einer der prominentesten Schweizer Ärzte und erster Zentralpräsident der schweizerischen Ärzteschaft – mit der Erarbeitung eines Gesetzesentwurfs. Im gleichen Zeitraum begann die Bundesverwaltung 1876 neben den seit 1867 erfassten Geburten, Sterbefällen und Trauungen auch Todesursachen zu erfassen und damit eine Mortalitätsstatistik aufzubauen (Historische Statistik der Schweiz 2012). Hier traf die Verwaltung bereits auf einige Herausforderungen – wie die unterschiedlichen Umsetzungen der einzelnen Kantone, die ärztliche Verwendung von Formularen – Todesscheinen in diesem Fall – sowie die Schwierigkeit der Diagnose, die ihr in der Folge auch bei der Erfassung der Morbiditätsstatistik basierend auf der Meldepflicht begegnen sollten.

Vor der Beschäftigung mit einem bundesstaatlichen Epidemiengesetz bestanden in den einzelnen Kantonen bereits Gesetzgebungen zur Prävention und Bekämpfung von ansteckenden Krankheiten, die sich jedoch teils stark unterschieden. Gerade bei der Meldepflicht rangierten die kantonalen Gesetze zwischen keiner Verpflichtung (Uri, Obwalden, Appenzell Innerrhoden und Genf) und einer für alle ansteckenden Krankheiten (Basel, Luzern, Zürich und Thurgau).^2^ Ein bundesweites Epidemiengesetz sollte diese Vielfalt an Gesetzgebungen und Meldewesen zumindest für bestimmte Krankheiten vereinheitlichen. Es stellte damit ein Harmonisierungsgesetz dar, das einen kleinen Teil der kantonalen Gesetze zusammenführen sollte. Dem schweizerischen Bund stand hier ausgehend vom Art. 69 der Verfassung die Bestimmung von Massnahmen gegen sogenannte „gemeingefährliche“ Krankheiten zu. Die Auswahl dieser Krankheiten wurde folgendermassen begründet:

Das kann aber kein anderes sein, als die Erkenntnis der Tatsache, dass es gewisse Seuchen gibt, bei denen die nach lokalen Bedürfnissen und Interessen gestaltete Gesetzgebung des einzelnen Kantons nicht ausreicht, ihre Verschleppung in andere Kantone zu verhüten; dass nur gemeinsame Vorkehrungen, die von allen Kantonen gleichmäßig zu treffen und stetig durchzuführen sind, das Land vor ihren Verheerungen zu bewahren vermögen. Seuchen dieser Art dürfen somit nicht nur lokale Bedeutung, sie müssen die entschiedene Tendenz zur Weiterverbreitung haben. Je stärker aber diese Tendenz hervortritt, je grösser die Verschleppbarkeit des Infektionsstoffes, je mehr derselbe Alle, einen Jeden bedroht, der mit ihm in Berührung kommt, je grösser die Neigung zu rascher sprungweiser Verbreitung durch das ganze Land, desto mehr sind wir berechtigt, die Epidemie eine „gemeingefährliche“ zu nennen.^3^

Die „gemeingefährlichen“ Krankheiten waren also solche, welche die Fähigkeit der Kantone, deren Ausbreitung mit ihren individuellen Gesetzgebungen zu unterbinden, überstiegen und so das ganze Land bedrohten. Diese waren für die eidgenössische Sanitätskommission Pocken, asiatische Cholera, Fleckfieber und Pest. Die Gruppe ist dabei nahezu identisch mit den gleichnamigen im deutschen Reichsseuchengesetz angegebenen Krankheiten; hier kam lediglich noch Lepra hinzu (Hüntelmann 2008: 196). Das Auswahlkriterium war dabei dasselbe: Für das kaiserliche Gesundheitsamt stellten diese Krankheiten grenzübergreifende Bedrohungen dar, weshalb gegen sie entsprechend einheitlich vorgegangen werden musste. Dieser Fokus der beiden Behörden muss jedoch mit der epidemiologischen Realität kontrastiert werden: Wie Beate Witzler gezeigt hat, hatten gerade die genannten großen Infektionskrankheiten aus einer mortalitätsstatistischen Perspektive bis Ende des 19. Jahrhunderts bereits stark an Relevanz verloren (Witzler 1995: 38f.). Die Behörden agierten demnach retrospektiv und sprachen mit den Gesetzen zu einem gewissen Maß bereits gelöste beziehungsweise regressive Probleme an. Im Entwurf des schweizerischen Epidemiengesetzes von 1879 hatte der Bund zusätzlich zu den „gemeingefährlichen“ Krankheiten die Berechtigung, gegen weitere Krankheiten Massnahmen zu ergreifen und entsprechend auch eine Meldepflicht einzuführen, sollten hohe Todeszahlen während eines Ausbruchs das begründen.^4^ Das war wichtig, da es dem schweizerischen Staat Spielraum gab, auf unerwartet große Ausbrüche von nicht „gemeingefährlichen“ Krankheiten oder neu auftretende Krankheiten zu reagieren.

Das von der eidgenössischen Sanitätskommission geplante bundesstaatliche Meldewesen sollte dabei zwei grundsätzliche Aufgaben erfüllen: Zum einen sollte es als Frühwarnsystem dienen, das beim Auftreten einer ansteckenden Krankheit den lokalen Behörden erlaubte, unmittelbar einschreiten zu können.^5^ Wie Axel Hüntelmann beschrieben hat, war dies der ursprüngliche Sinn der Medizinalstatistik: die Erfassung des gesundheitlichen Zustands eines Staates und seiner Bevölkerung (Hüntelmann 2019: 32). Das Feststellen der Erkrankungen – also eine grundlegende epidemiologische Untersuchung zum Krankheitsgeschehen – beschrieb die Sanitätskommission als Ursprung jeglicher seuchenpolizeilicher Maßnahmen (Vögele 2014: 30). Zum anderen sah sie in der schweizweiten Sammlung der epidemischen Krankheitsfälle die Möglichkeit, statistische Informationen von hohem wissenschaftlichem Wert zu erfassen. Die Bestimmung von Fällen in Abhängigkeit von ihrem zeitlichen und örtlichen Verlauf sollte Erkenntnisse zur Ätiologie der Krankheiten erlauben – mit der Hoffnung der Sanitätskommission, dass sie daraufhin praktisch in der öffentlichen und privaten Hygiene verwendet würden und in Zukunft die Bekämpfung der Krankheiten verbessern könnten.^6^

In den Folgejahren wurde der Gesetzesentwurf der Sanitätskommission vom Ständerat, der Kantonsvertretung, und dem Nationalrat, der Volksvertretung, diskutiert sowie überarbeitet und Anfang 1882 verabschiedet (Ruckstuhl & Ryter 2017: 120). Damit wäre das schweizerische Epidemiengesetz nach Ablauf der Referendumsfrist in Kraft getreten. Jedoch enthielt das Gesetz eine bundesweite Impfpflicht gegen Pocken. Diese war in der Schweiz auf Bundes- und Kantonsebene zuvor bereits – ähnlich wie in anderen europäischen Ländern zu der Zeit – heftig diskutiert worden (Nebe et al. 2021: 101; Wolff 1996: 79; Huerkamp 1985: 628). Ultimativ hatten sich die eidgenössische Sanitätskommission, der Ständerat und der Nationalrat für die Aufnahme der Impfpflicht in das Epidemiengesetz entschieden. Im Frühjahr 1882 mobilisierten sich jedoch die Impfgegner:innen und sammelten die benötigten Referendumsunterschriften, um eine Volksabstimmung über die Einführung des Epidemiengesetzes auszulösen. Dabei wurde der vorgeschlagene Gesetzestext im Juli 1882 von der Bevölkerung deutlich abgelehnt.

Das staatliche und kantonale Verlangen nach einem bundesweiten Epidemiengesetz blieb jedoch bestehen, weshalb bis 1886 ein weiterer, stark gekürzter Entwurf erarbeitet wurde. Er enthielt keine Impfpflicht und wurde von beiden Räten ohne große Änderungen angenommen. Auch wurde diesmal kein Referendum angestrebt, und ab dem 2. Juli 1886 trat das Gesetz in Kraft.^7^ An der Meldepflicht selbst hatte sich seit dem ersten Entwurf wenig geändert. Das revidierte Epidemiengesetz als Gesamtes wies jedoch einen zentralen Unterschied zur Fassung von 1879 auf: Es bezog sich ausschließlich auf die „gemeingefährlichen“ Krankheiten und enthielt für den Bund keine Möglichkeit, situativ auch andere Krankheiten als solche einzustufen. Vermutlich beschränkte er selbst seine Kompetenz im Gesetz, um eine erfolgreiche Einführung desselben sicherzustellen. Damit bestand ab 1886 eine bundesweite Meldepflicht nur für die vier „gemeingefährlichen“ Krankheiten und keine Möglichkeit, sie auf andere Krankheiten auszuweiten. Die Kompetenz über jegliche Bekämpfung von nicht „gemeingefährlichen“ Krankheiten – unabhängig davon, ob sie sich auch über Kantonsgrenzen hinweg ausbreiteten – und die entsprechenden Bestimmungen über die Meldepflicht lagen also weiterhin bei den einzelnen Kantonen.

Ab 1886 war im Schweizer Bundesstaat also ein Epidemiengesetz in Kraft, das für vier Krankheiten überkantonal die Prävention und Bekämpfung bestimmte. Entsprechend konnte die bundesstaatliche Meldepflicht nur für diese Krankheiten ihre beiden Aufgaben des Frühwarnsystems und der statistischen Erfassung während einer Epidemie erfüllen. Doch wie erfolgte in einem stark föderalistisch geprägten Land wie der Schweiz die Umsetzung des Epidemiengesetzes und der staatlichen Meldepflicht? Sie wurde wiederum an die Kantone delegiert, die mithilfe von Vollziehungsverordnungen die Gestaltung des Gesetzes bestimmten. Damit hatten sie für ihr Hoheitsgebiet einen gewissen Spielraum, wie die staatlichen Vorgaben zu interpretieren seien. Bezogen auf die Meldepflicht hatte das etwa Einfluss darauf, wer in welcher Situation zur Meldung verpflichtet war, wem gemeldet wurde, da sanitätspolizeiliche Aufgaben in den verschiedenen Kantonen unterschiedlich organisiert wurden, und welche zeitlichen Fristen für die Meldungen bestanden.^8^

Für das nachfolgende Fallbeispiel eines Pockenausbruchs in Schaffhausen trat die Vollziehungsverordnung des Epidemiengesetzes und damit die dortigen Bestimmungen über die staatliche Meldepflicht am 25. September 1889 in Kraft.^9^ Die Verordnung verpflichtete in erster Linie den/die Inhaber:in eines Hauses, in dem der Verdacht einer Infektion mit den vier „gemeingefährlichen“ Krankheiten bestand, dies dem Gemeinderatspräsidenten oder einem hierfür ausgewählten Mitglied der örtlichen Gesundheitskommission mündlich, schriftlich oder telegrafisch anzuzeigen. Im Fall der Abwesenheit oder Erkrankung des/der Hausbesitzers/:in fiel diese Meldepflicht jedem/r volljährigen Hausgenossen:in zu. Die Verpflichtung von Lai:innen, Krankheitsfälle zu melden, war in der Schweiz schon seit dem 17. Jahrhundert häufig Teil der Seuchenbekämpfung.^10^ Auch in den während des 19. Jahrhunderts eingeführten Seuchengesetzen in anderen europäischen Ländern wurden Lai:innen zur Meldung von Krankheitsfällen verpflichtet.^11^ Das führte zu einer zentralen Schwierigkeit in der Umsetzung der Meldepflicht: dem Identifizieren von Krankheitsfällen. Ob Lai:innen dazu fähig waren, wurde im ersten Entwurf des schweizerischen Epidemiengesetzes,^12^ sowie auch bei der Erarbeitung des deutschen Reichseuchengesetzes von 1900 angezweifelt (Hess 2009: 312f.). Beide Gesetzestexte enthalten die Lai:innenmeldung jedoch in ihrer angenommenen Form. Im Epidemiengesetz war der Grund dafür, dass vergangene Epidemieerfahrungen gezeigt hatten, welche Folgen die Verheimlichung von Fällen durch Angehörige mit sich brachten. Gewissermaßen war das in Anbetracht der Diagnoseschwierigkeiten ein notwendiges Übel der Seuchengesetzgebung, welches die Ärzte trotz kritischer Position tolerierten. Das Nichtbeachten dieser Bestimmungen konnte mit Geldbußen bestraft werden. In Schaffhausen waren in zweiter Instanz alle praktizierenden, eidgenössisch befähigten Ärzte dazu verpflichtet, der Meldung der Betroffenen – in der Vollziehungsverordnung als „Hilferuf“ bezeichnet – Folge zu leisten und nach Bestimmung des „Krankheitscharakters“ den Bezirksarzt, den Gemeinderatspräsidenten oder den Präsidenten der Gesundheitskommission auf schnellstem Weg in Kenntnis zu setzen. Daraufhin war der Bezirksarzt verpflichtet, sich persönlich vor Ort ein Bild der Lage zu machen, die notwendigen Vorkehrungen zu treffen und der Sanitätsdirektion des Kantons Bericht zu erstatten sowie die einzelnen Fälle zu melden.

Der Kanton Schaffhausen delegierte also die erste Aufgabe der Meldepflicht als Frühwarnsystem an Lai:innen wie Hausbesitzer:innen beziehungsweise auch an alle behandelnden Ärzte. Sie hatten den Behörden das Auftreten von Krankheitsfällen möglichst umgehend mitzuteilen, damit sie daraufhin entsprechende Massnahmen ergreifen konnten. In der Folge war der Bezirksarzt im Sinne der zweiten Aufgabe verpflichtet, für die statistische Erfassung der Krankheitsfälle eine definitive Diagnose zu stellen. Die Erfüllung der ersten Aufgabe basierte auf Verdacht und Krankheitskenntnis der entsprechenden Personen (Brown 1995: 43f.). Dementsprechend konnte sie auch basierend auf Vorwissen oder Anleitungen von Lai:innen ausgeführt werden.^13^ Eine offene Frage ist hier, ob sich die meldepflichtigen Lai:innen ihrer Pflicht auch bewusst waren. Die zweite Aufgabe des Bezirksarztes, eine eindeutige Aussage über den Krankheitsfall zu treffen, die anschließend wissenschaftlich verarbeitet werden sollte, war sehr viel schwieriger und gerade um 1900 bei Infektionskrankheiten immer noch eine Herausforderung. Da diese zweite Aufgabe die Grundlage der Seuchenstatistiken ist, interessiere ich mich in dieser Studie primär für ihre Umsetzung und besonders dafür, wie genau die Identifikation und Kommunikation der jeweiligen Krankheiten ablief.

Die schweizerische Epidemiengesetzgebung und das Meldewesen waren nach 1886 also zwar durch die staatliche Gesetzgebung vorgegeben, doch die Umsetzung und Gestaltung lag in den Händen der Kantone. Zusätzlich beschränkten sich die staatlichen Vorgaben nur auf die vier rar gewordenen „gemeingefährlichen“ Krankheiten. Ob und wie mit weiteren ansteckenden Krankheiten verfahren wurde, konnten die Kantone frei entscheiden. Um ein Verständnis der praktischen Umsetzung des schweizerischen Meldewesens aufzubauen, muss daher neben einem Fallbeispiel einer der „gemeingefährlichen“ Krankheiten auch ein Beispiel einer kantonal meldepflichtigen Krankheit untersucht werden. Der folgende Abschnitt untersucht deshalb zuerst den Pockenausbruch in Schaffhausen 1903 und daraufhin einen Typhusausbruch im Kanton Luzern 1904, um aufzuzeigen, wie ein Kanton die Meldepflicht für nicht „gemeingefährliche“ Krankheiten organisierte.

## Umsetzung der Meldepflicht für „gemeingefährliche“ Krankheiten

Der Pockenausbruch in Schaffhausen im Jahr 1903 bietet sich hierbei als Fallbeispiel an, da zwei im Archiv erhaltene Berichte des dortigen Bezirksarztes einen Einblick in seine Meldepraxis geben.^14^ Auch stellt der Ausbruch die Art von Situation dar, für die das Epidemiengesetz und die Meldepflicht ausgelegt war: Ausbrüche von zwar „gemeingefährlichen“ Krankheiten, die jedoch dank der Meldepflicht auf eine kleinräumige Verbreitung begrenzt werden konnten. Im nachfolgenden Beispiel interessiere ich mich vor allem für die Interaktionen zwischen dem Bezirksarzt und seinen Patient:innen, die zur Stellung der Diagnose, zur Meldung und damit zur Aufnahme in die Krankheitsstatistik geführt haben. Durch die geringe Anzahl an Fällen, die beim Pockenausbruch in Schaffhausen 1903 festgestellt wurden, sind die Beschreibungen der einzelnen Fälle und der Diagnosepraxis des Bezirksarztes in den Berichten sehr detailliert und erlauben es somit, die Durchführung seiner Meldepraxis nachzuvollziehen.

Die beiden Berichte sind Dokumente, die der Bezirksarzt der Stadt Schaffhausen, Dr. Emil Rahm senior, am 13. April und am 2. Juni 1903 an die Sanitätsdirektion des Kantons Schaffhausen schrieb. Emil Rahm wurde 1837 geboren, schloss 1859 an der Universität Zürich sein Medizinstudium ab und war ab 1873 bis zu seinem Tod 1909 36 Jahre lang Bezirksarzt in Schaffhausen (Universität Zürich 2022). In seinen Berichten beschreibt Dr. Rahm einen zusammenhängenden Ausbruch von Pocken in Stadt und Umgebung Schaffhausen, in dem er den ersten Fall am 19. März und den letzten am 17. April 1903 feststellte. Insgesamt erwähnt Emil Rahm in seinen Berichten 15 Pockenfälle. Von allen Fällen außer einem erfuhr der Bezirksarzt durch behandelnde Ärzte. Sie kontaktierten ihn mit einem Verdacht auf Pocken. Obwohl also im Kanton Schaffhausen die Haushaltsvorstände zur Meldung von Krankheitsfällen verpflichtet waren, teilten im Pockenausbruch von 1903 nahezu ausschließlich behandelnde Ärzte sie dem Bezirksarzt mit. Das ist naheliegend, da im städtischen Schaffhausen die medizinische Versorgung gut war und Ärzte daher tendenziell vor dem eindeutigen Ausformen einer meldepflichtigen Krankheit mit den Patient:innen Kontakt hatten. Aus diesem Grund ist wahrscheinlich in den Quellen auch nichts über rechtliche Konsequenzen bei Nichtmeldung für die Haushaltsstände vorhanden. Nach der Meldung stand die Frage der Diagnose, die daraufhin in die Statistik einfloss, aber noch aus: Dr. Rahm begab sich nach der Mitteilung der behandelnden Ärzte zu der betroffenen Person und war mit der Herausforderung konfrontiert: Wie diagnostizierte man 1903 eindeutig einen Pockenfall?

In erster Linie versuchte Dr. Rahm, die Verdachtsfälle basierend auf den individuellen Krankheitssymptomen zu bestimmen. Eine grundlegende Herausforderung hierbei war die Unterscheidung zwischen den ähnlich auftretenden Variola (Pocken) und Varicellen (Windpocken). Das wurde nochmals erschwert durch das unterschiedliche Auftreten der Krankheiten je nach individueller Erkrankung. Auch konnten sich eigentlich gegen Pocken geimpfte Personen dennoch infizieren, meist hatten sie jedoch einen leichten Verlauf mit wenigen Symptomen, was die Diagnose wiederum erschwerte. Das führte beim Bezirksarzt in gewissen Fällen zu Unsicherheiten, die Krankheit nur basierend auf den Symptomen festzustellen. Aus diesem Grund nutzte Emil Rahm verschiedene andere Wege, um eine Entscheidung zu treffen und diese zu begründen.

Die ersten Fälle des Pockenausbruchs in Schaffhausen wurden bei einer gewissen Familie Buchter festgestellt. Bei seinem ursprünglichen Besuch am 17. März 1903 fand Dr. Rahm bereits eine „massenhafte Eruption“ von kleinen Stippchen bei Robert Buchter vor, die er aber aufgrund der geringen Größe der Bläschen und dem Ausbleiben der Vorläufersymptome einer Pockenerkrankung (Kopfweh, Schüttelfrost, Fieber und „bedeutendes kaum erträgliches Rückenweh“) für einen Varicellenfall hielt.^15^ Zwei Tage später besuchte er die Familie erneut und hätte rein basierend auf den Symptomen von Robert Buchter ihn mit Windpocken diagnostiziert, wenn nicht seine Schwester zur gleichen Zeit an den Vorläufersymptomen gelitten und auch einen kleinen Ausschlag entwickelt hätte. In einem anderen Bericht an den Stadtrat Schaffhausen vom 27. März betont Dr. Rahm, wie wichtig dieser zweite, typische Fall für das Feststellen der Pocken beim ersteren, atypischen war.^16^ Daraufhin diagnostizierte er beide mit Pocken und meldete das. Hier half der Kontext der darauffolgenden Erkrankungen, die symptomatischen Unklarheiten aufzulösen. Gleichzeitig bedeutete es, dass für die eindeutige Identifikation von Krankheitsfällen mehr als nur ihre individuellen Merkmale bewertet wurden.

Auch setzte der Bezirksarzt bei der Feststellung der Pockenerkrankungen auf seine Berufserfahrung: Der behandelnde Arzt der Familie Buchter, Dr. Moser, konsultierte Dr. Rahm, um seine Vermutung zu bestätigen, die auch für ihn „beim ersten Besuch zweifelhaft war, obschon ich [Dr. Rahm] schon über 100 Pockenfälle in Behandlung & Beobachtung hatte“.^17^ Durch die wiederholte Erfahrung mit der Erkrankung erhoffte sich Emil Rahm, die Fälle identifizieren zu können. Auch in den Diskussionen zur Meldepflicht im deutschen Reichsseuchengesetz war eine Sorge, dass einem Teil der Ärzte die meldepflichtigen Krankheiten noch nie begegnet waren, weshalb eine Diagnose für sie schwierig sei (Hess 2009: 312). Hier spielte die Erfahrung des einzelnen diagnostizierenden und meldenden Arztes mit der jeweiligen Krankheit eine wichtige Rolle, um die entsprechenden Nuancen einer Erkrankung zu erkennen und sie von ähnlichen unterscheiden zu können. Das war jedoch aufgrund des seltenen Auftretens gerade der „gemeingefährlichen“ Krankheiten um 1900 schwierig, und selbst wenn diese Erfahrung vorhanden war, half sie – wie hier – teils nicht weiter.

Zusätzlich zu den Symptomen, dem Kontext der individuellen Erkrankung und der Erfahrung des Arztes hatte auch die gegenwärtige epidemische Lage einen Einfluss auf die Diagnose. Dr. Rahm beschreibt in seinem Bericht an die Sanitätsdirektion vom 13. April 1903:

Bekanntlich ist das Erkennen von eigentlichen Pocken in den Anfangsstadien kaum möglich und oft, wenn eine Anzahl Fälle bekannt sind, oder eine Epidemie in der Nähe ist, können Pocken schon im Anfangen diagnostiziert [werden], weil dann der Gedanke an eine derartige Krankheit so rasch bei jedem Arzte, wie auch bei den Laien schon beim ersten Auftreten von kleinen Stippchen im Gesichte, von Hals und am Körper Verdacht erregt.^18^

Ob Fälle von Pocken in der Umgebung gemeldet wurden, hatte also einen direkten Einfluss darauf, wie darauffolgende Krankheitsfälle eingeschätzt wurden. Dr. Rahm bezieht sich bei seiner Entscheidung, eine Erkrankung als Varicellen oder Variola zu kategorisieren, mehrmals spezifisch auf die gegenwärtige Pockenlage. In deutschen Anweisungen zur Pockenbekämpfung von 1904 wird explizit beschrieben, dass, sobald die frühen Anzeichen einer Erkrankung unter Umständen vorkommen, die Pocken befürchten lassen (wie etwa während einer Epidemie), dies den Verdacht bereits vor Ausbruch des charakteristischen Ausschlags und anderer Symptome zulasse (Kaiserliches Gesundheitsamt 1904). Dementsprechend sei die Erkrankung auch zu melden. In ihrer Untersuchung von Caesar Adolf Bloeschs ärztlichen Journalen beobachtet Lina Gafner die gleiche Anpassung der Diagnosepraxis und Bezugnahme auf anderen Krankheitsfälle während einer Epidemie (Gafner 2016: 261). Dabei erfasst sie, dass Bloesch während einer Epidemie in Biel 1837 zu Beginn keine Diagnose bei seinen Patient:innen notierte, aber nach einigen Wochen in seinen Journalen klar von Ruhrfällen sprach. Das habe möglichweise mit der offiziellen Bezeichnung der Epidemie als Ausbruch von Ruhr durch die Regierung zu tun gehabt.

Diese Herausforderungen des Bezirksarztes bei der definitiven Diagnose von Infektionskrankheiten und seine Lösungsansätze zeigen, dass Meldungen und entsprechend historische Statistiken zu Krankheitsvorkommen mehr sind als nur objektive Darstellungen von Erkrankungen. Aus diesem Grund argumentiere ich, dass man sie nicht nur als Repräsentationen von Krankheitsfällen, sondern auch als medizinisch-soziale Interaktionen der meldenden und erkrankten Person sowie ihrer Umwelt ansehen und entsprechend historisieren muss. Historische Statistiken stellen Zusammenfassungen von Tausenden solcher Interaktionen und individuellen Interpretationen von Personen in amtlichen Positionen wie Dr. Rahm dar, die in spezifischen Situationen und Kontexten mit den beschriebenen praktischen Herausforderungen entstanden sind. Das problematisiert die Aussagekraft der Meldungen und Statistiken, da die Meldungen auf einer instabilen Symptomatologie entsprechend unterschiedlichen Krankheitsvorstellungen und Erfahrungen der meldenden Person, individuellen Kontexten von Erkrankungen sowie Epidemiesituationen basierten. Damit verlieren die Statistiken ihre scheinbare Schärfe bei der Einteilung und Bezifferung von Krankheitsfällen. Bei einer geschichtswissenschaftlichen Verwendung von historischen Statistiken muss das dementsprechend in Betracht gezogen werden.

Neben der Aufnahme der beobachteten Fälle in die Morbiditätsstatistik hatten die Meldungen auch epidemiebekämpfende Maßnahmen zur Folge. Ausgehend von den ersten Meldungen wurde Dr. Rahm vom Stadtrat beauftragt, Massenimpfungen in der Bevölkerung durchzuführen. Ob es dagegen Proteste gab, ist nicht klar. Widerstand regte sich vor allem gegen die vom Bezirksarzt ausgesprochene Isolation und Quarantäne, die jegliche Bewohner:innen von pockenverseuchten Häusern betraf. Der Grund für den Widerstand war vor allem die dadurch ausgelöste zwangsläufige Unterbrechung der Erwerbstätigkeit, wobei solche Einkommensverluste vom Kanton Schaffhausen in quittierten Ausgleichszahlungen – weiteren wichtigen *paper technologies* – ersetzt wurden. Der einzige – archivarisch erhaltene – Widerstand gegenüber der Meldung selbst war der letzte in der Pockenepidemie von 1903 erfasste Fall von Jakob Gubler, welcher versuchte, seine Erkrankung zu verheimlichen. Er wurde Dr. Rahm von dessen Vorarbeiter mit der Mitteilung, er sei seit zwei Tagen vom Arbeitsplatz weggeblieben und seine Nebenarbeiter würden vermuten, er sei pockenkrank, gemeldet und daraufhin besucht sowie diagnostiziert. Ob es weitere solche – erfolgreichere – Fälle der Vertuschung einer Erkrankung gab, ist verständlicherweise nicht archivarisch bestimmbar, aber wahrscheinlich.

Dieses Fallbeispiel hat die Umsetzung der Meldepflicht für eine „gemeingefährliche“ Krankheit illustriert. Wie bereits beschrieben, stellte es jedoch nur einen Bruchteil des schweizerischen Meldewesens dar, das zwar von Staatsseite vorgegeben war, jedoch von den Kantonen gestaltet wurde. Ob und wie Bestimmungen über alle nicht „gemeingefährlichen“ Krankheiten bestanden, lag vollständig in den Händen der Kantone, die entsprechend unterschiedlich damit umgingen. Das anschließende Fallbeispiel beschäftigt sich daher mit einem Typhusausbruch im Kanton Luzern von 1904, der beispielhaft für die Funktionsweise der 25 kantonalen vorgegebenen Meldewesen stehen soll. Der Typhusausbruch ist dabei für die Untersuchung des schweizerischen Meldewesens auch interessant, da für Typhus zu diesem Zeitpunkt aus wissenschaftlicher Perspektive die oben beschriebene Herausforderung der Diagnose einer Infektionskrankheit um 1900 – zumindest in der Theorie – gelöst war.

## Umsetzung einer kantonalen Meldepflicht

Typhus war eine der Infektionskrankheiten, bei der zu Beginn des 20. Jahrhunderts ein Feststellen des Krankheitserregers mithilfe laboratorischer Methoden möglich war. Mithilfe des 1896 entwickelten Gruber-Widal-Tests konnte durch sero-diagnostische Agglutination – also einen Bluttest – das Vorkommen der Erreger in gewissen Fällen laboratorisch festgestellt werden (Linton 2000: 104). Für die Wissenschaft stabilisierte sich dadurch das Krankheitskonzept, da Typhus nicht mehr basierend auf Symptomen und in Abgrenzung von diversen Differenzialdiagnosen bestimmt werden musste. Im nachfolgenden Fallbeispiel des Typhusausbruchs im Kanton Luzern kommen solche Tests jedoch bei der Feststellung der Krankheitsfälle nicht vor. Zu diesem Zeitpunkt wäre die Durchführung des Gruber-Widal-Tests in der Schweiz an der Untersuchungsstation des Instituts zur Erforschung der Infektionskrankheiten der Universität Bern höchstwahrscheinlich bereits möglich gewesen (Institut zur Erforschung der Infektionskrankheiten 1907). Die Gründe für das Fehlen der Tests im Luzerner Typhusausbruch sind wahrscheinlich beim damit verbundenen Aufwand und den Kosten zu suchen sowie an den unterschiedlichen laboratorischen Möglichkeiten der einzelnen Kantone (Hardy 1993: 52). Es wäre im Allgemeinen und gerade bei einem kleinen Ausbruch sehr ungewöhnlich gewesen, eine mit einer ansteckenden Krankheit infizierte Person von einem ländlichen Teil des Kantons Luzern nach Bern zu schicken. Eine Durchführung des Gruber-Widal-Tests durch die meldenden Ärzte selbst war höchstwahrscheinlich nicht möglich, da ihnen die Zeit und das Equipment für eine sero-diagnostische Agglutination fehlten. Auch ist denkbar, dass sie weiterhin auf ihre Fähigkeit, Infektionskrankheiten über Symptome und andere Anzeichen zu diagnostizieren, setzten – also ähnlich wie Dr. Rahm auf ihre vorherige Erfahrung mit der Krankheit vertrauten. Das zeigt, dass wissenschaftliche Entwicklungen nicht umgehend in medizinische Praxis einflossen (Taddei 2010: 36, 101; Worboys 2006).

Die Gestaltung des Meldewesens durch die Kantone erlaubte die Einführung anderer Innovationen, die den Meldeprozess und die damit verbundenen Herausforderungen erleichtern sollten. Sie waren zu Beginn des 20. Jahrhunderts vor allem von administrativer Natur. Im Unterschied zum Kanton Schaffhausen wurden in Luzern vorgedruckte Meldeformulare eingesetzt, die den Prozess und die damit gesammelten Informationen vereinheitlichten. Die Formulare waren wichtige Arbeitsdokumente und *paper technologies*, die meist nach Eingang bei den Sanitätsbehörden zusammengefasst und entsorgt wurden, weshalb sie in Archiven selten erhalten sind. Bei den Archivquellen des Typhusausbruchs von 1904 ist das der Fall, weshalb sich eine Untersuchung desselben hier anbietet. Nachfolgend wird untersucht, ob diese Innovation bei der Bewältigung der grundlegenden Herausforderungen der Meldepflicht, illustriert im ersten Fallbeispiel, weiterhalfen und wie sich diese administrative Praxis auf den Inhalt der Meldungen auswirkte.

Der Kanton Luzern bietet sich hierbei auch für eine Betrachtung der Meldepraxis an, da er bereits vor Einführung des Epidemiengesetzes einer der Kantone mit einer umfangreichen Meldepflicht war und gerade auch zu Beginn des 20. Jahrhunderts Wert auf den Aufbau von Kontroll- und Aufsichtsorganen legte (Galliker 2013: 163). Im gesamtschweizerischen Kontext illustriert das, welche Möglichkeiten bei der Gestaltung der Meldepflicht und des Epidemiengesetzes bestanden. In Luzern waren im Unterschied zu Schaffhausen für die Früherkennung von Krankheitsfällen keine Lai:innen, sondern nur praktizierende Ärzte meldepflichtig, die gleichzeitig auch die Diagnose für die Seuchenstatistik stellten.^19^ Damit war eine größere Anzahl von Ärzten im Kanton Luzern bei der statistischen Erfassung von Infektionskrankheiten beteiligt, die mit den Meldeformularen die von ihnen beobachteten Fälle kommunizierten. Für diese Studie stellt sich ausgehend davon die Frage, wie sich solche anderen normativen Vorgaben auf die Meldepraxis auswirkten und welche Rolle die Verwendung von Meldeformularen dabei spielte. Oberflächlich betrachtet, versprach es eine breitere Abdeckung des Meldewesens und eine standardisierte Sammlung von statistischen Informationen. Ob das auch in der Praxis der Fall war, zeigt die nachfolgende Betrachtung der Meldepraxis.

Die erste Meldung des Luzerner Typhusausbruchs von 1904 traf am 10. Januar bei der Ortsgesundheitskommission Ebersecken ein.^20^ Dabei waren zwei Personen aus der Familie Marti bereits am 20. Dezember 1903, eine weitere Ende Dezember und eine vierte am 10. Januar erkrankt, wobei erst der letzte Fall zum Einsatz eines Arztes und damit zur Meldung geführt hatte. Ungefähr einen Monat später wurden weitere Fälle von Typhus in der Region gemeldet sowie von Gerüchten berichtet, dass die Krankheit bei mehreren dort wohnhaften Familien ausgebrochen sei. Daraufhin wurden der Amtsarzt des Amtes Willisau, Dr. Cyrill Kaufmann, sowie sein Stellvertreter Dr. Alfred Rösli beauftragt, dem nachzugehen und einen Bericht anzufertigen. Ihre Untersuchungen vor Ort bestätigten die Gerüchte und sie meldeten diverse weitere Fälle von Typhus. Das illustriert nochmals die Herausforderung der ursprünglichen Bestimmung von Krankheitsfällen – gerade in ländlichen Regionen –, wenn Haushaltsvorstände nicht zur Meldung verpflichtet waren. Wurde keine medizinische Hilfe aufgesucht, konnten Fälle nicht oder – wie beim Typhusausbruch im Kanton Luzern – nur durch alternative Kanäle wie Gerüchte bestimmt werden.

**Figure 1:**
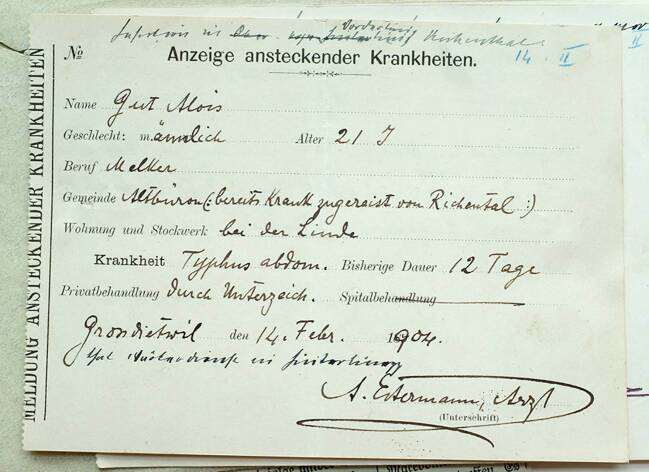

Die Meldungen der Ärzte erfolgten dabei alle über die gesetzlich vorgegebenen Meldeformulare. Sie haben ungefähr das Format einer Postkarte und wurden in perforierten Heftern vom Kanton an die meldepflichtigen Ärzte herausgegeben. Sie sollten einen Teil des Meldeprozesses und damit auch die Sammlung von Informationen für die schweizerische Seuchenstatistik standardisieren. Der Kanton Luzern verlangte dabei die Angabe von allgemeinen Personendaten der Erkrankten wie Name, Geschlecht, Alter, Beruf und Wohnort, aber auch diverse Informationen über die Erkrankung sowie ihren Verlauf (Abb. 1 und 2).

**Figure 2:**
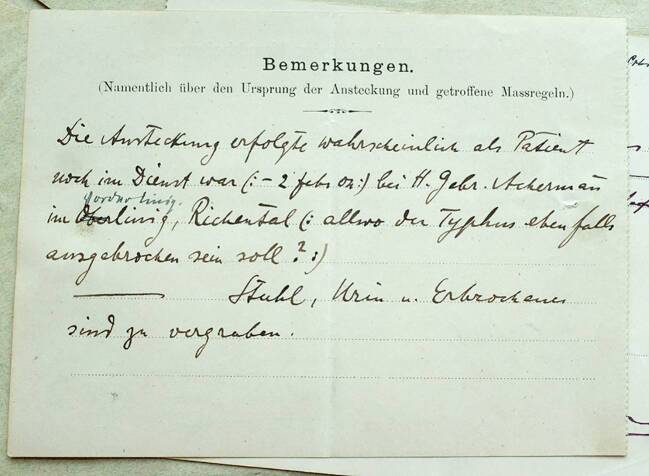

In den Formularen werden dabei ähnliche Herausforderungen beim Melden sichtbar wie im ersten Fallbeispiel. Die eindeutige Diagnose von Typhus basierend auf Symptomen war schwierig, da es diverse Differenzialdiagnosen gab (Steere-Williams 2020: 8). In medizinischen Lehrbüchern Ende des 19. Jahrhunderts wurde Abdominaltyphus zum einen von anderen typhusartigen Krankheiten wie Flecktyphus und „Rückfallsfieber“ abgegrenzt, zum anderen gab es auch innerhalb der Diagnose diverse leichte, mittelschwere und schwere Krankheitsverläufe, die sich unterschiedlich ausbildeten (Fleischer 1888: 37, 49). Auf den Meldeformularen ist bei gewissen Fällen wie beispielsweise dem des Hans Marti neben der Diagnose ein Fragezeichen notiert.^21^ Dieser Fall war einer der ersten, der im Rahmen des Typhusausbruchs 1904 gemeldet wurde und der vierte innerhalb derselben Familie. Basierend auf dem individuellen Kontext der Erkrankung wäre eine Diagnose mit Typhus daher naheliegend gewesen, doch musste es Kriterien gegeben haben, welche die Zweifel des meldenden Arztes begründeten. In der Statistik der entsprechenden Woche sind nur die drei Fälle der anderen Familienmitglieder aufgelistet, die vor Hans Marti erkrankt waren – dementsprechend wurde der Zweifelsfall nicht aufgenommen (Schweizerisches Gesundheitsamt 1904).

Eine wichtige Kategorie auf den Meldeformularen in Anbetracht des im ersten Fallbeispiel entwickelten Verständnisses von Meldungen als medizinisch-soziale Interaktionen ist die Angabe der „bisherigen Dauer“ der Erkrankung. Auf gewissen Formularen des Luzerner Typhusausbruchs ist sie auch mit der Kategorie „Tag der Erkrankung“ angegeben. Dabei existieren bei den einzelnen Meldungen große Unterschiede. Die frühsten Angaben sind identisch mit der Datierung der Meldung selbst, die längsten umfassen bis zu drei Wochen zwischen dem vermuteten Beginn der Erkrankung und der Anzeige derselben. Diese zeitlichen Unterschiede hatten zum einen mit dem Verlauf der Erkrankung zu tun: Beim Fall von Alois Gut (Abb. 1 und 2) konnte während des ersten Besuchs von Dr. Estermann am 3. Februar „eine sichere Diagnose […] noch nicht gestellt werden“.^22^ Sein Verdacht war, dass es sich entweder um eine schwere Influenza oder Typhus abdominalis handle. Erst in der zweiten Woche der Erkrankung war er sich sicher, dass Alois Gut an Typhus erkrankt war und machte dementsprechend zu diesem Zeitpunkt die Meldung.

Bei den späteren Fällen des Typhusausbruchs, die erst durch die Nachforschungen des Amtsarztes und dessen Stellvertreter bestimmt werden konnten, verzögerte sich die Meldung, da die Betroffenen keine medizinische Hilfe aufsuchten. Ohne die Untersuchung hätte keine im Kanton Luzern meldepflichtige Person von ihrer Erkrankung erfahren. Das war der Nachteil einer Gesetzgebung, die keine verpflichtende Lai:innenmeldung enthielt: Der Meldung sowie damit der statistischen Erfassung wurde in den meisten Fällen der Einsatz eines Arztes vorausgesetzt. Wurde kein Arzt eingesetzt, blieben die Krankheitsfälle für beide Zielsetzungen der Meldepflicht – der Früherkennung, um umgehend eine Verbreitung zu verhindern, und der statistischen Erfassung der Erkrankungen aus wissenschaftlichen Gründen – unentdeckt. Beim Luzerner Typhusausbruch von 1904 konnten durch die Nachforschungen weitere Betroffene bestimmt werden, allerdings zeitlich verzögert.

Darin zeigt sich, dass die Verschriftlichung der Krankheitsfälle durch die Meldeformulare und die Statistiken einen zentralen Einfluss auf den Informationsgehalt derselben hatten. Durch die Meldung wurden die Krankheitsfälle mit einem bestimmten Zeitpunkt verbunden, jedoch nicht basierend auf dem vermuteten Beginn der Erkrankung, sondern nach der Datierung des Meldeformulars selbst und damit nach der oben angesprochenen medizinisch-sozialen Interaktion, welche aus der Diagnose und Meldung hervorging. Dadurch zeigt sich ein zentraler Unterschied zwischen dem Verlauf des Krankheitsausbruchs und der verschriftlichten Repräsentation desselben: Der dynamische Epidemieverlauf wird durch die Meldung und Verarbeitung in Statistiken in statische Schriftlichkeit umgewandelt. Für ein geschichtswissenschaftliches Verständnis greift es daher zu kurz, die Statistiken nur als objektive Darstellung anzusehen. Vielmehr ist das, was sie abbilden, der Akt des Meldens, der – wie in den Fallbeispielen des Pockenausbruchs und des Typhusausbruchs sichtbar war – von diversen, teils nicht-medizinischen Faktoren beeinflusst wurde.

Die Fallbeispiele der bundesstaatlichen und kantonalen Umsetzung der schweizerischen Meldepflicht haben gezeigt, wie komplex die Produktionsprozesse der Meldungen und Seuchenstatistiken schon bei Krankheitsausbrüchen waren, die einen geografisch beschränkten Raum und eine überschaubare Anzahl Fälle umfassten. Mit ihren zwei Zielsetzungen war die Meldepflicht grundsätzlich für diese Situationen ausgelegt und – trotz den beschriebenen Schwierigkeiten – grösstenteils erfolgreich. Die Spanische Grippe stellte eine neue Herausforderung für dieses Meldewesen dar. Die Influenza gehörte im Sommer 1918 nicht zu den „gemeingefährlichen“ und damit schweizweit meldepflichtigen Krankheiten. Nur einige Kantone verlangten ihre Meldung. Wie die Einführung des schweizerischen Epidemiengesetzes in den 1880er Jahren gezeigt hatte, waren Änderungen an der bundesstaatlichen Gesetzgebung zeitintensiv und aufwendig; das Gegenteil der Ausbreitungsfähigkeit der Influenza. Was passierte nun, als dieses schweizerische Meldewesen auf die Spanische Grippe traf?

## Melden während der Spanischen Grippe ab 1918

Die schweizerische Meldepflicht wurde im Vorfeld der Spanischen Grippe von verschiedenen Seiten hinterfragt. Bereits 1909 forderten diverse Kantone eine Erweiterung der Gruppe der „gemeingefährlichen“ Krankheiten durch Aufnahme der epidemischen Genickstarre, vor allem damit diese besser als durch die freiwillige Meldung der einzelnen Kantone an den Bund statistisch erfasst werde.^23^ 1913 wurde der dem Epidemiengesetz zugrunde liegende Bundesverfassungsartikel Art. 69 revidiert, um dem Bund zu ermöglichen, Bestimmungen gegen Tuberkulose zu erlassen.^24^ Zuvor bezog sich dieser explizit nur auf die „gemeingefährlichen“ Krankheiten. In der revidierten Version der Verfassung fiel diese Bezeichnung weg und wurde durch „übertragbare oder stark verbreitete oder bösartige Krankheiten“ ersetzt.^25^ Damit hatte der Schweizerische Bund ab 1913 neu die Fähigkeit, nicht nur gegen Tuberkulose, sondern gegen alle ansteckenden Krankheiten Massnahmen zu bestimmen. Eine darauffolgend diskutierte Erneuerung des Epidemiengesetzes wurde durch den Ersten Weltkrieg unterbrochen, weshalb noch bis zur Revision von 1921 von „gemeingefährlichen“ Krankheiten gesprochen wurde.

Mit dem Ausbruch des Ersten Weltkriegs am 28. Juli 1914 und dem Ausrufen des extrakonstitutionellen Staatsnotrechts in der Schweiz am 3. August, welches dem Bundesrat erlaubte, ohne Bestätigung der Räte Gesetzesanpassungen durchzuführen, folgte als Teil der Kriegsmaßnahmen eine temporäre Verschärfung der Meldepflicht für ansteckende Krankheiten (Schneider 2019: 14f.). Obwohl die Schweiz keine Kriegspartei war, führte der Kriegsausbruch dennoch zur Mobilisierung der Armee, die auch entsprechende Herausforderungen in der Seuchenprävention verursachte. Auch litt sie in der zweiten Hälfte des Krieges unter ausbleibenden Nahrungsmittelimporten. Am 27. Oktober 1914 wurde die Gruppe der „gemeingefährlichen“ Krankheiten um Typhus abdominalis, Paratyphus, Scharlach, Diphterie, epidemische Genickstarre und epidemische Poliomyelitis anterior acuta erweitert.^26^ Die Begründung dahinter war, dass diese Krankheiten durch die Kriegssituation zu Bedrohungen geworden waren, welche die kantonalen Kompetenzen überstiegen. Daher müssten diese nach geltendem Epidemiengesetz als „gemeingefährlich“ angesehen und in der ganzen Schweiz vereinheitlicht gegen sie vorgegangen werden. In den Folgejahren wurden im Kriegsverlauf dieser Gruppe noch weitere Krankheiten – unter anderem ab Herbst 1918 die Influenza – hinzugefügt.

Die Spanische Grippe brach in der Schweiz im Sommer 1918 aus. Die ersten Influenzameldungen, die das schweizerische Gesundheitsamt nachträglich bereits als Teil der Pandemie ansah, wurden Mitte Mai angezeigt (Schweizerisches Gesundheitsamt 1919). Ursprünglich wurde dies nicht als außergewöhnlich angesehen und es kam in der letzten Juniwoche schweizweit kein einziger Grippefall zur Meldung (Sonderegger 1991: 25). Erst in der ersten Juliwoche wurden über das ganze Land verteilt verschiedene Ausbrüche der Influenza gemeldet. Zu diesem Zeitpunkt stiegen auch die Mortalitätsraten, weshalb Christian Sonderegger und Andreas Tscherrig argumentieren, hier den Anfangspunkt der Spanischen Grippe in der Schweiz zu setzen (Sonderegger & Tscherrig 2016: 268).

Kurz nach dem Auftreten dieser Krankheitsherde in der Schweiz sowie im Kontext der Mitteilungen von anderen europäischen Ländern ersuchte das schweizerische Gesundheitsamt am 6. Juli 1918 die kantonalen Sanitätsdirektionen, dieses mit wöchentlichen Rapporten über Zahl und Verbreitung der Fälle sowie über den Charakter der Krankheit zu informieren.^27^ Auch sollten die Ärzte ihre Beobachtungen für eine wissenschaftliche Verarbeitung – ähnlich der zweiten Aufgabe der Meldepflicht – zusammenstellen. Diese Bitte führte dazu, dass in gewissen Kantonen daraufhin eine offizielle Meldepflicht für die Influenza eingeführt wurde. Beispielsweise nahm der Kanton Zürich am 25. Juli 1918 die Influenza in die *Verordnung betreffend die Bekämpfung von übertragbaren Krankheiten* auf.^28^ Diese verpflichtete alle Ärzte, beobachtete Krankheitsfälle der Gesundheitsbehörde des Wohnorts mitzuteilen. Andere Kantone wie etwa Solothurn sahen hingegen von einer Meldepflicht für Krankheitsfälle ab, da diese für die Ärzte eine zu große Belastung zusätzlich zur medizinischen Versorgung der Erkrankten darstellen würde, und verlangten nur die umgehende Meldung von mit der Spanischen Grippe in Zusammenhang stehenden Todesfällen und eine periodische Orientierung über Erkrankungen.^29^

Während der ersten Welle der Spanischen Grippe in der Schweiz, deren Höhepunkt zwischen Juli und August 1918 war, lag die Gestaltung der Meldepflicht für Influenza also in den Händen der Kantone. Damit verhielt sich dieses Meldesystem ähnlich wie das für die nicht „gemeingefährlichen“ Krankheiten nach 1886. Die Kantone bestimmten, ob und wie eine Meldepflicht für die Spanische Grippe umgesetzt werden sollte, und kommunizierten ihre Erkenntnisse daraufhin dem Bund. Wie an der Gegenüberstellung von Zürich und Solothurn erkennbar ist, waren die Unterschiede zwischen den einzelnen Meldesystemen teils entsprechend groß.

Nachdem die Anzahl Fälle und Tode aufgrund der Spanischen Grippe Anfang September in der Schweiz abgeflaut war, stieg sie zum Ende des Monats erneut rasch und stark an (Sonderegger 1991: 26). Als Reaktion darauf führte der Bund am 11. Oktober 1918 mit Verweis auf die Bundesverfassungsrevision von 1913 und den Vollmachten vom 3. August 1914 eine schweizweite Meldepflicht für die Influenza ein, indem er diese unter die Gesetzgebung für „gemeingefährliche“ Krankheiten stellte (Schweizerisches Gesundheitsamt 1918a). Jedoch beschränkte er die Meldepflicht nur auf Ärzte und umfasste nicht die im Epidemiengesetz aufgelisteten Haushaltsvorstände, da bei der Diagnose von Influenza oft „sehr große Untersicherheit“ bestünde (Schweizerisches Gesundheitsamt 1918b). Auch bezog sich die Verpflichtung der umgehenden Meldung nur auf die ersten in einem Ort auftretenden Erkrankungen, um den bereits überlasteten Ärzte keinen allzu großen Mehraufwand aufzutragen. Darauffolgende Fälle sollten in einem wöchentlichen Rapport mitgeteilt werden. In gewissen Kantonen führte dies – zumindest auf Papier – zu einer Lockerung des Meldewesens – so etwa im Kanton Zürich; in anderen zu einer Verschärfung desselben – wie etwa im Kanton Solothurn, der neu dazu verpflichtet war, Neuerkrankungen umgehend zu melden.

Dieses System blieb jedoch nicht lange bestehen. An der interkantonalen Grippekonferenz vom 5. November 1918 kam von diversen kantonalen Sanitätsbehörden die Mitteilung, dass beim Auftreten von zahlreichen Neuerkrankungen es den mit Arbeit überlasteten Ärzte nicht möglich sei, alle Personendaten der Erkrankten zu erfassen und damit zu melden.^30^ Aus diesem Grund verlangten sie, nur eine summarische Anzeige der Grippefälle erstatten zu dürfen – wie während den ersten Grippemonaten. Das schweizerische Gesundheitsamt entschied daraufhin, dass die Kantone nur zusammenfassende Wochenanzeigen einreichen können, sofern sie der Ansicht seien, man könne auf genauere Influenzameldungen verzichten. Knapp drei Wochen nach Einführung der bundesweiten Meldepflicht für Influenza sah das Gesundheitsamt also von einer strikten Durchführung desselben ab und überließ die Bestimmung der Meldepflicht für Influenza wieder den Kantonen. Dies ist exemplarisch für die Funktionsweise schweizerischer Staatsorgane, die zwar zentralisierte Entscheidungen trafen, jedoch die Verantwortung für die Umsetzung – selbst während des Höhepunkts einer Krise wie der Spanischen Grippe – den Kantonen überließen. Die praktische Umsetzung der Meldepflicht für Influenza war mit den gleichen Herausforderungen konfrontiert, die bereits in den Vorjahrzehnten ein genaues statistisches Erfassen von ansteckenden Krankheiten erschwerte. Zusätzlich wurden die bestehenden Herausforderungen des Meldewesens durch die grassierende Epidemie intensiviert.

Die eindeutige Diagnose der Spanischen Grippe war aufgrund des unterschiedlichen Auftretens der Krankheit schwierig. Wissenschaftlich bestand zu Beginn des 20. Jahrhundert keine eindeutige Krankheitskonzeption der Grippe (Vagneron 2021). Zwar existierten bald nach Ausbruch der Spanischen Grippe 1918 in der Schweiz offizielle Beschreibungen des Krankheitsbilds, die entsprechend auch den Ärzte kommuniziert wurden, doch halfen diese nur bedingt weiter.^31^ Ähnlich wie im Fallbeispiel des Pockenausbruchs in Schaffhausen 1903 wurde bei der Diagnose der Epidemiekontext miteinbezogen. Dies wurde jetzt jedoch auch kritisiert: Rudolf Staehelin, Professor für innere Medizin an der Universität Basel, sah im Oktober 1918 das Feststellen der pandemischen Influenza als schwierig an, wenn diese nicht im Zusammenhang mit einer Epidemie auftrete (Staehelin 1919). Die Unterscheidung mit den jährlich auftretenden „auf unspezifischer Infektion beruhenden fieberhaften Affektionen“ sei unmöglich, weshalb aufgrund der aktuellen Epidemielage die Diagnose Grippe „oft zu leicht gestellt“ werde. Eine Reflexion über mögliche Auswirkungen davon auf die Statistiken zur Spanischen Grippe unternahm Staehelin nicht. Geschichtswissenschaftlich ist dies auch ein Aspekt, der beispielsweise von Wilfried Witte bei den deutschen Statistiken zur Influenza diskutiert wurde, bei der jegliche Todesfälle durch Pneumonien der Grippe zugewiesen wurden (Witte 2006: 321). Wie Krankheitsfälle in der Statistik eingeteilt werden sollten, war eine Herausforderung, die in der Vergangenheit auch vom wissenschaftlichen Erkenntnisprozess ausgelöst wurde: Beim Aufbau der deutschen Medizinalstatistik in den 1870er Jahren wurden beispielsweise durch die Entdeckung des Tuberkuloseerregers die Kategorien „Lungenschwindsucht“, „Knochenschwund“ und „Skrofulose“ in der Statistik zu „Tuberkulose“ zusammengefasst oder – die umgekehrte Entwicklung – die sogenannten syphilitischen Erkrankungen durch die Entdeckung der Erreger in individuelle Krankheiten wie „Syphilis“, „Gonorrhö“ oder „Ulcus molle“ aufgeteilt (Hüntelmann 2019: 37).

Häufig traten aber bei der Meldung der Influenzafälle auch Verzögerungen auf. Die Überlastung der Ärzte führte während der Epidemie zu einer zwangsläufigen Vernachlässigung von administrativen Aufgaben. Aus diesem Grund wurde – wie oben beschrieben – die umgehende Meldung von Krankheitsfällen auf Neuausbrüche beschränkt und summarische Berichte über darauffolgende Fälle wurden nur wöchentlich verlangt. Trotzdem mussten die kantonalen Sanitätsbehörden die Ärzte regelmäßig ermahnen, diese rechtzeitig einzureichen.^32^ Aus Sicht der historischen Akteur:innen war das größte Problem bei der genauen Erfassung der Influenzafälle in der Schweiz die zwangsläufige Beschränkung der Meldepflicht auf Ärzte. Bereits im Juli 1918 schätzten die meldenden Ärzte, dass von den erkrankten Personen nur etwa jede zweite medizinische Hilfe aufsuchte und somit erfasst wurde.^33^ Gerade leichte Fälle wurden wenig erfasst, da solche Patient:innen keine ärztliche Behandlung in Anspruch nahmen.^34^ Aus diesem Grund ist anzunehmen, dass schwere Fälle einen verhältnismäßig übergroßen Anteil der gemeldeten Fälle und damit der Influenzastatistik ausmachten. Ähnlich stellte sich dies auch bei der Erfassung von Dysenterie im Deutschen Kaiserreich dar, bei der – obwohl nur fünf Fälle den Behörden gemeldet wurden – in einer darauffolgenden Studie mithilfe von Bluttests bei 130 von 628 Personen sowohl der Erreger als auch Antikörper gegen diesen bestimmt wurden. Diese Personen hatten wahrscheinlich lediglich milde Symptome, sodass sie keine medizinische Unterstützung aufsuchten (Linton 2010: 613).

Damit bestand bei der Spanischen Grippe gleichzeitig eine Verzerrung, die man im heutigen epidemiologischen Vokabular als „overreporting“ bezeichnen würde – durch die von Staehelin beschriebene Zuweisung von unspezifischen Infektionen zur Grippe –, sowie auch eine, die man als ein „underreporting“ verstehen kann – durch die Überlastung der Ärzte und ihren geringeren Kontakt mit leichten Erkrankungen. Die diagnostischen Schwierigkeiten bei der Grippe führten wahrscheinlich sowohl zu falsch positiven wie auch falsch negativen Diagnosen, wobei gerade schwere und typische Verläufe in der Statistik im Verhältnis zur Gesamtzahl übermäßig auftraten. Das ist ein Aspekt, der auch in der zeitgenössischen medizinischen Diskussion über die Influenzastatistiken – die sich jedoch in der Schweiz grösstenteils in Grenzen hielt – spezifisch angesprochen wurde, weshalb diese vom Professor für Innere Medizin an der Universität Bern, Hermann Sahli, als „nie ganz richtig“ bezeichnet wurden (Sahli 1919: 4). Meine Vermutung ist, dass die Unsicherheit einer Morbiditätsstatistik allgemein und gerade während einer Epidemie für die historischen Akteure – meldende Ärzte wie staatliche Behörden – zu einem gewissen Maß selbstverständlich war und sich deshalb in den schweizerischen Publikationen wie dem *Korrespondenzblatt für Schweizer Ärzte* und dem *Bulletin des Schweizerischen Gesundheitsamtes* keine breitere Diskussion findet.

Eine weitere Auswirkung der Spanischen Grippe auf das Meldewesen war eine Reduktion des Informationsinhalts der Meldungen. Die wöchentlichen Berichte an das schweizerische Gesundheitsamt umfassten nur noch die Anzahl gemeldeter Fälle für die entsprechenden Gemeinden und Ämter, teilweise unterschieden in leichte und schwere Verläufe. Eine Sammlung von genauen wissenschaftlichen Informationen im Sinne der zweiten Zielsetzung der Meldepflicht, die der Bund im Juli 1918 von den Kantonen verlangt hatte, war damit nicht möglich. Zurückblickend waren sich die historischen Akteur:innen dessen auch bewusst. Im Geschäftsbericht des Schweizerischen Gesundheitsamts für das Jahr 1918 heißt es explizit, dass es mit der Einführung der bundesweiten Meldepflicht ab dem 11. Oktober nicht das Ziel hatte, alle beobachteten Fälle zur Anzeige zu bringen (Schweizerisches Gesundheitsamt 1919). Das sei unmöglich gewesen und hätte bei der großen Verbreitung der Krankheit auch nicht viel genutzt. Vielmehr sei das Ziel gewesen, den Verlauf der Epidemie zu verfolgen und rechtzeitig vom Auftreten der Krankheit in zuvor verschonten Gemeinden zu erfahren.

Die Meldepraxis während der Spanischen Grippe sollte daher losgelöst von den zwei ursprünglichen Zielsetzungen derselben angesehen werden. Eine Früherkennung im Sinne des ersten Ziels sowie auch die statistische Erfassung für wissenschaftliche Zwecke im Sinne der zweiten Zielsetzung war nicht möglich. Vielmehr führten die großflächige Epidemiesituation und ihre Auswirkungen auf die administrative Praxis zu einer Simplifizierung der Meldungen und Statistiken, die nur noch grobe Aussagen darüber ermöglichten, wann wo wie viele Fälle auftraten. Christian Sonderegger erachtet die Morbiditätsdaten in Schweiz für die Spanische Grippe deshalb als unbrauchbar für eine geschichtswissenschaftliche Untersuchung und verwendet sie in seiner Arbeit nicht (Sonderegger 1991: 23). Wie diese Studie gezeigt hat, könnte die angemessene Historisierung der Meldungen und Statistiken eine Lösung des Problems sein. Dies erklärt dadurch auch, weshalb während der Spanischen Grippe in der Schweiz benachbarte und demografisch ähnliche Gemeinden teils sehr unterschiedliche Fall- und Todeszahlen meldeten (Nussbaum 1982: 244). Eine Betrachtung der Schnittstelle zwischen den normativen Vorgaben und den historischen Statistiken ist dabei zentral, um zu beleuchten, was die Gesetzestexte effektiv regulierten, und auch um zu verstehen, welche Limitationen die historischen Statistiken haben.

## Fazit: Das Melden als praktische Herausforderung

Infolge der Erfahrungen mit der bundesweiten Meldepflicht seit ihrer Einführung 1886 erfolgte 1921 eine Revision sowohl was ihren Umfang als auch was den Ablauf betraf. Die vier ursprünglich als „gemeingefährlich“ identifizierten Krankheiten Pocken, Cholera, Fleckfieber und Pest rückten – nachdem sie bereits vor Einführung des Epidemiengesetzes grösstenteils an Bedeutung verloren hatten – weiter in den Hintergrund. Die verschärfte Meldepflicht während des Ersten Weltkriegs und die Spanische Grippe hatten gezeigt, dass die Prävention und Bekämpfung von zusätzlichen Krankheiten ein schweizweites Vorgehen verlangten. Auch verdeutlichte die Notlage ab 1914, dass die Epidemienbekämpfung von einer größeren Entscheidungsfreiheit und Flexibilität des Bundes profitierte. Aus diesem Grund wurde am 18. Februar 1921 der Bund mit einer Revision des Epidemiengesetzes dazu ermächtigt, dieses – sollten es die Umstände verlangen – auch auf andere Krankheiten auszuweiten (Schweizerisches Gesundheitsamt 1921a). Das war bereits im ersten Entwurf des Gesetzes von 1879 enthalten gewesen, der jedoch 1882 abgelehnt wurde – was unter anderem zur retrospektiven Orientierung des Epidemiengesetzes geführt hatte. In Anbetracht der Aufhebung des Vollmachtenregimes und der zeitgleich erfolgten Verschärfung des Meldewesens beschloss der Bundesrat am 23. August 1921, die zwischen 1914 und 1921 im Notrecht hinzugefügten Krankheiten in das Epidemiengesetz aufzunehmen (Schweizerisches Gesundheitsamt 1921b). Damit waren nunmehr 16 Krankheiten bundesweit meldepflichtig, unter anderem die Influenza. Auch waren von da an für die Früherkennung in erster Instanz behandelnde Ärzte meldepflichtig und Lai:innen nur dann, sofern sie keine ärztliche Hilfe aufsuchten. Hier legte der Bund also mehr Wert auf präzise Aussagen über die Krankheitsfälle als auf eine breite Abdeckung. Gleichzeitig haben die beiden Fallstudien von Schaffhausen und Luzern gezeigt, dass Lai:innen in der Praxis ohnehin nur selten aktiv im Meldeprozess involviert waren. Das schweizerische Meldewesen nach 1921 führte die ursprünglichen beiden Zielsetzungen der Meldepflicht weiter, erhöhte aber den Umfang der zu meldenden Krankheiten und die Flexibilität des Bundes, auf zukünftige Notlagen zu reagieren. Die Umsetzung lag aber weiterhin grösstenteils bei den Kantonen. Das verdeutlicht, dass bei der Existenz von übergreifenden Gesetzen wie dem schweizerischen Epidemiengesetz und parallelen lokalen Bestimmungen wie den kantonalen Vollziehungsbestimmungen genau betrachtet werden muss, was sie regulieren und wie sie in der Praxis umgesetzt wurden. Wie die Studie gezeigt hat, sind Probleme nicht gelöst, selbst wenn sie auf einer normativen Ebene gelöst scheinen.

Die hier erfolgte Betrachtung des schweizerischen Meldewesens zwischen 1886 und 1921 hat die praktischen Herausforderungen der Umsetzung eines solchen Systems gezeigt. Für die Seuchengeschichte illustriert das auf einer methodischen Ebene die Historizität der für die Statistiken verwendeten Meldungen und zeigt zusätzlich, dass ein Blick hinter die Zahlen – auf ihre Herstellungsprozesse – eine reiche Einsicht in die medizinische Praxis gibt und Statistiken als geschichtswissenschaftliche Quellen verkompliziert. Krankheitsdiagnosen – einzelne wie auch gesammelte – sind Produkte ihrer Zeit und müssen als solche historisiert werden. Meldungen dokumentieren nicht nur die Krankheitsfälle, sondern repräsentieren die medizinisch-sozialen Interaktionen, aus denen sie entstehen. Die Verschriftlichung von Erkrankungen durch die Meldung zeitigt den dynamischen Verlauf einer Krankheit sowie damit einer Epidemie und produziert ein statisches Abbild davon. Diese Aspekte von Fallzahlen und Morbiditätsstatistiken müssen bei ihrer geschichtswissenschaftlichen Verwendung als Quellen oder grundlegendes Datenmaterial berücksichtigt werden. Ein Weg kann sein, wie in dieser Studie die praktischen Produktionsprozesse der Statistiken näher zu betrachten. Gleichzeitig öffnen sich dadurch auch die medizinisch-sozialen Interaktionen als Untersuchungsgegenstände, an denen Erkenntnisse über die Diagnose von Infektionskrankheiten um 1900, über Ärzte in staatlichen Positionen und die Diffusion von wissenschaftlichem Wissen erlangt werden können (Worboys 2006). Die Diagnose von Pocken war zu Beginn des 20. Jahrhunderts immer noch eine Herausforderung und von diversen, teils nicht-medizinischen Faktoren abhängig. Wissenschaftliche Fortschritte in der Bakteriologie und ihre Aufnahme in die medizinische Praxis bei der Feststellung von Krankheiten wie Typhus verliefen nicht gleichzeitig und verlangen eine nähere Betrachtung der spezifischen Möglichkeiten der historischen Akteur:innen. Andere Innovationen wie die Verwendung von Meldeformularen zur Standardisierung der Informationssammlung wurden um 1900 eingesetzt, die als *paper technologies *einen eigenen, nicht zu vernachlässigenden Einfluss auf das Meldewesen hatten. Epidemien wie die Spanische Grippe strapazieren neben der medizinischen Versorgung auch administrative Systeme wie die Meldepflicht; dies war jüngst auch bei COVID-19 sichtbar. Aus einer geschichtswissenschaftlichen Perspektive hat die Studie somit gezeigt, dass quantitative Quellen wie Statistiken gemeinsam mit qualitativen wie Meldeformularen und ärztlichen Berichten der Historiker:innen nicht nur einen Überblick des Umfangs und der Entwicklung von Krankheitsausbrüchen erlauben, sondern auch einen Einblick in zentrale medizinische Prozesse der Vergangenheit.

## Footnotes

1 Obwohl es während der untersuchten Zeitperiode eine kleinere Anzahl Ärztinnen in der Schweiz gab, tauchen sie in den untersuchten Quellen nicht auf, weshalb in diesem Artikel die rein männliche Bezeichnung verwendet wird. Die Erwähnungen von „Ärzten“ in Bezug auf die gesetzlichen Regelungen beziehen sich verständlicherweise auf den gesamten Berufsstand.

2 Schweizerisches Bundesarchiv (BAR) E87#1000/1166#17*, Botschaft des Bundesrats an die hohe Bundesversammlung, betreffend den Bundesgesetzentwurf über Einrichtungen und Massnahmen zur Verhütung und Bekämpfung gemeingefährlicher Epidemien, vom 18. Dezember 1879, S. 22.

3 Botschaft des Bundesrats an die hohe Bundesversammlung, betreffend den Bundesgesetzentwurf, vom 18. Dezember 1879, S. 7.

4 BAR E87#1000/1166#17*, Bundesgesetz betreffend Massnahmen gegen gemeingefährliche Epidemien, vom 31. Januar 1882.

5 Botschaft des Bundesrats an die hohe Bundesversammlung, betreffend den Bundesgesetzentwurf, vom 18. Dezember 1879, S. 19.

6 Botschaft des Bundesrats an die hohe Bundesversammlung, betreffend den Bundesgesetzentwurf, vom 18. Dezember 1879, S. 22.

7 BAR E87#1000/1166#21*, Bundesgesetz betreffend Massnahmen gegen gemeingefährliche Epidemien vom 2. Juli 1886.

8 BAR E87#1000/1166#4*, Kantonale Vollziehungsverordnungen zum eidgenössischen Epidemiengesetz, 1888–1891.

9 BAR E87#1000/1166#4*, Kantonale Vollziehungsverordnung zum Bundesgesetz betreffend Massnahmen gegen gemeingefährliche Epidemien vom 2. Juli 1886, vom 25. September 1889.

10 Siehe beispielsweise Staatsarchiv Zürich: III AAb 1.4, Nr. 97, Mandat der Stadt Zürich betreffend Verhalten bei Seuchen und Meldepflicht der Pfarrer, vom 28. Oktober 1668.

11 Botschaft des Bundesrats an die hohe Bundesversammlung, betreffend den Bundesgesetzentwurf, vom 18. Dezember 1879, S. 20.

12 Ebd.

13 Diese Anleitungen beschrieben die Merkmale und den zeitlichen Verlauf einer Pockenerkrankung in für Lai:innen verständlicher Sprache. Ein solches Beispiel ist: Anweisung zur Bekämpfung der Pocken. Festgestellt in der Sitzung des Bundesrats vom 28. Januar 1904. Besondere Beilage zu den „Veröffentlichungen des Kaiserlichen Gesundheitsamtes“. Berlin: Verlag von Julius Springer: 24/25.

14 Stadtarchiv Schaffhausen (StaSH): C II.27.01.03.02/03, Der Bezirksarzt des Bezirks Schaffhausen an die Sanitätsdirektion Schaffhausen, vom 13. April 1903; Der Bezirksarzt des Bezirks Schaffhausen an die Sanitätsdirektion Schaffhausen, vom 2. Juni 1903.

15 StaSH C II.27.01.03.02/03, Der Bezirksarzt des Bezirks Schaffhausen an die Sanitätsdirektion Schaffhausen, vom 13. April 1903.

16 StaSH C II.27.01.03.02/03, Der Bezirksarzt des Bezirks Schaffhausen an den Stadtrat von Schaffhausen, vom 27. März 1903.

17 StaSH C II.27.01.03.02/03, Der Bezirksarzt des Bezirks Schaffhausen an den Stadtrat von Schaffhausen, vom 27. März 1903.

18 StaSH C II.27.01.03.02/03, Der Bezirksarzt des Bezirks Schaffhausen an die Sanitätsdirektion Schaffhausen, vom 13. April 1903.

19 BAR E3300A#1000/761#112*, Verordnung betreffend Massnahmen gegen die Verbreitung epidemischer Krankheiten, welche nicht dem Bundesgesetz vom 2. Juli 1886 unterstellt sind, vom 9. Dezember 1903.

20 Staatsarchiv Luzern (StaLU) AKT 44/1890, Meldeformular von Hans Marti, vom 10. Januar 1904.

21 StaLU AKT 44/1890, Meldeformular von Hans Marti, vom 10. Januar 1904.

22 StaLU AKT 44/1890, Dr. Estermann an den Amtsarzt Dr. Kaufmann, vom 25. Februar 1904.

23 BAR E3300A#1000/761#112*, Das eidg. Departement des Innern an die kantonalen Sanitätsbehörden, vom 22. Februar 1909.

24 BAR E3300A#1000/761#6*, Motion Rickli betr. Verfassungsrevision, vom 18. Oktober 1909.

25 In der Verfassungsrevision von 1913 wurden nur bei gewissen Artikeln Änderungen vorgenommen. Die Verfassung basierte weiterhin auf der Version von 1874. Auf der bei der Literaturangabe der Bundesverfassung von 1874 angegebenen Webseite sind diese Änderungen vermerkt.

26 BAR E27#1000/721#19693*, Bundesratsbeschluss betreffend Ausdehnung der in Art. 3 des Bundesgesetzes über Massnahmen gegen gemeingefährliche Epidemien vorgeschriebenen Anzeigepflicht, vom 27. Oktober 1914.

27 StaLU AKT44/1898, Der Direktor des Schweizerischen Gesundheitsamt an den Herrn Sanitätsrat des Kantons Luzern, vom 6. Juli 1918. Diese Mitteilung ging an alle Sanitätsdirektionen der Schweiz.

28 BAR E3300A#1000/761#89*, Verfügung der Direktion des Gesundheitswesens zuhanden der Gesundheitsbehörden, Bezirksärzte und Ärzte des Kantons Zürich betreffend Anzeigepflicht der Grippe, vom 25. Juli 1918.

29 BAR E3300A#1000/761#89*, Das Sanitäts-Departement des Kantons Solothurn an das Schweizerische Gesundheitsamt, vom 23. Juli 1918.

30 BAR E3300A#1000/761#89*, Kreisschreiben an die Mitglieder des Sanitäts-Kollegiums und die Ärzte des Kantons Solothurn über die ärztliche Meldepflicht betreffend die Spanische Krankheit (Grippe), vom 6. Dezember 1918.

31 BAR E3300A#1000/761#89*, Kreisschreiben an die Ärzte und örtlichen Gesundheitsbehörden betreffend die Grippe-Epidemie, vom 17. Juli 1918.

32 Kreisschreiben an die Mitglieder des Sanitäts-Kollegiums betreffend die Spanische Krankheit (Grippe), vom 6. Dezember 1918.

33 StaLU AKT44/1898, J. Mattmann an die Sanitätskanzlei des Kantons Luzern, vom 24. Juli 1918.

34 StaLU AKT44/1906, Der Sanitätsrat des Kantons Luzern an das Polizeidepartement desselben, vom 23. Oktober 1919.

## References

[CR1] Belich J (2022). The world the plague made: the Black Death and the rise of Europe.

[CR2] Brown, Phil 1995. Naming and Framing: The Social Construction of Diagnosis and Illness. *Journal of Health and Social Behavior *(Extras Issue): 34–52. 7560848

[CR3] Bryder L (1988). Below the magic mountain: a social history of tuberculosis in twentieth-century Britain.

[CR4] Bundesamt für Gesundheit 2022. Coronavirus: Massnahmen und Verordnungen. URL: https://www.bag.admin.ch/bag/de/home/krankheiten/ausbrueche-epidemien-pandemien/aktuelle-ausbrueche-epidemien/novel-cov/massnahmen-des-bundes.html#1570431754 (1.2.2023).

[CR5] Bundesverfassung der Schweizerischen Eidgenossenschaft, vom 12. September 1848. URL: https://www.verfassungen.ch/verf48-i.htm (1.2.2023).

[CR6] Bundesverfassung der Schweizerischen Eidgenossenschaft, vom 29. Mai 1874. URL: https://www.verfassungen.ch/verf74-i.htm (1.2.2023).

[CR7] Cunningham A (2002). Identifying disease in the past: cutting the Gordian knot. Asclepio.

[CR8] Fichter, Adrienne 2020. „Die Zahl der Todesfälle haben wir aus Wikipedia entnommen“. *Republik* (online 20.3.2020). URL: https://www.republik.ch/2020/03/20/die-zahl-der-todesfaelle-haben-wir-aus-wikipedia-entnommen (1.2.2023).

[CR9] Fleischer R (1888). Lehrbuch der inneren Medizin für Studierende und Ärzte.

[CR10] Gafner L, Dinges M (2016). Administrative and Epistemic Aspects of Medical Practice: Caesar Adolf Bloesch (1804–1863). Medical Practice, 1600–1900.

[CR11] Galliker H-R, Hürlimann K (2013). Staat und Verwaltung – Aufbau, Ausbau und Reformen. Der Kanton Luzern im 20. Jahrhundert.

[CR12] Haas S, Schneider MC, Bilo N (2019). Die Zählung der Welt. Kulturgeschichte der Statistik vom 18. bis 20. Jahrhundert.

[CR13] Hardy A (1993). The epidemic streets: infectious disease and the rise of preventive medicine, 1856–1900.

[CR14] Hess B-J (2009). Seuchengesetzgebung in den deutschen Staaten und im Kaiserreich vom ausgehenden 18. Jahrhundert bis zum Reichsseuchengesetz 1900.

[CR15] Historische Statistik der Schweiz 2012. *Krankheiten und Todesursachen: Einleitung*. URL: https://hsso.ch/de/2012/d (15.08.2023).

[CR16] Hüntelmann AC (2008). Hygiene im Namen des Staates. Das Reichsgesundheitsamt 1876–1933.

[CR17] Hüntelmann AC, Haas S (2019). Konstruktion und Etablierung der Medizinalstatistik in Deutschland, ca. 1850–1900. Die Zählung der Welt. Kulturgeschichte der Statistik vom 18. bis 20. Jahrhundert.

[CR18] Hüntelmann AC, Falk O (2021). Accounting for Health. Calculation, paperwork, and medicine, 1500–2000.

[CR19] Huerkamp C (1985). The history of smallpox vaccination in Germany: a first step in the medicalization of the general public. Journal of Contemporary History.

[CR20] Institut zur Erforschung der Infektionskrankheiten (1907). Anleitung zur Entnahme und Einsendung von Material zum Zweck bakteriologischer Untersuchung.

[CR21] Janssens A, Devos I (2022). The limits and possibilities of cause of death categorisation for understanding late nineteenth century mortality. Social History of Medicine.

[CR22] Jorland G, Weisz G, Jorland G, Opinel A, Weisz G (2005). Introduction: who counts?. Body counts. Medical quantification in historical and sociological perspective.

[CR23] Kaiserliches Gesundheitsamt (1904). Anweisung zur Bekämpfung der Pocken. Festgestellt in der Sitzung des Bundesrats vom 28. Januar 1904.

[CR24] Kavey R-EW, Kavey AB (2021). Viral pandemics: from smallpox to COVID-19.

[CR25] Leven KH (1997). Die Geschichte der Infektionskrankheiten. Von der Antike bis ins 20. Jahrhundert.

[CR26] Leven KH, Paul N, Schlich T (1998). Krankheiten: Historische Deutung versus retrospektive Diagnose. Medizingeschichte: Aufgaben, Probleme, Perspektiven.

[CR27] Linton DS (2000). Was typhoid inoculation safe and effective during world war I? debates within German military medicine. Journal of the History of Medicine and Allied Sciences.

[CR28] Linton DS (2010). “War Dysentery” and the Limitations of German Military Hygiene during World War I. Bulletin of the History of Medicine.

[CR29] Mol A (2002). The body multiple. Ontology in medical practice.

[CR30] Mooney G (2015). Intrusive interventions: public health, domestic space, and infectious disease surveillance in England, 1840–1914.

[CR31] Nebe J, Schwanke E, Groß D (2021). The influence of epidemics on the concept of the Bogeyman. Historical Social Research.

[CR32] Nussbaum W (1982). Die Grippe-Epidemie 1918–1919 in der schweizerischen Armee. Gesnerus.

[CR33] Reinhardt V (2021). Die Macht der Seuche. Wie die Große Pest die Welt veränderte, 1347–1353.

[CR34] Ruckstuhl B, Ryter E (2017). Von der Seuchenpolizei zu Public Health. Öffentliche Gesundheit in der Schweiz seit 1750.

[CR35] Sahli H (1919). Über die Influenza. Korrespondenzblatt für Schweizer Ärzte.

[CR36] Schneider O (2019). Die Schweiz im Ausnahmezustand. Expansion und Grenzen von Staatlichkeit im Vollmachtenregime des Ersten Weltkriegs, 1914–1919.

[CR37] Schweizerisches Gesundheitsamt (1904). Ansteckende Krankheiten. Sanitarisch-demographisches Wochenbulletin der Schweiz.

[CR38] Schweizerisches Gesundheitsamt (1918). Bundesratsbeschluss betreffend Ausdehnung der Anzeigepflicht für gemeingefährliche Krankheiten auf die Influenza, vom 11. Oktober 1918. Bulletin des Schweizerischen Gesundheitsamtes.

[CR39] Schweizerisches Gesundheitsamt (1918). Kreisschreiben des Schweizerischen Gesundheitsamtes an die kantonalen Sanitätsbehörden betreffend die Massnahmen gegen die Influenza (obligatorische Anzeige usw.), vom 15. Oktober 1918. Bulletin des Schweizerischen Gesundheitsamtes.

[CR40] Schweizerisches Gesundheitsamt (1919). Bericht des Schweizerischen Gesundheitsamtes über seine Geschäftsführung im Jahre 1918. Bulletin des Schweizerischen Gesundheitsamtes.

[CR41] Schweizerisches Gesundheitsamt (1921). Bundesgesetz betreffend Abänderung des Bundesgesetzes vom 2. Juli 1886 betreffend Massnahmen gegen gemeingefährliche Epidemien. Bulletin des Schweizerischen Gesundheitsamtes.

[CR42] Schweizerisches Gesundheitsamt (1921). Bundesratsbeschluss betreffend die Anzeigepflicht für übertragbare Krankheiten. Bulletin des Schweizerischen Gesundheitsamtes.

[CR43] Sonderegger C (1991). Die Grippeepidemie 1918/19 in der Schweiz.

[CR44] Sonderegger C, Tscherrig A, Krämer D, Pfister C, Segesser DM (2016). Die Grippepandemie 1918–1919 in der Schweiz. „Woche für Woche neue Preisaufschläge“. Nahrungsmittel‑, Energie- und Ressourcenkonflikte in der Schweiz des Ersten Weltkrieges.

[CR45] Staehelin R (1919). Einige Bemerkungen über die klinischen Erscheinungen und die Diagnose der pandemischen Influenza. Korrespondenzblatt für Schweizer Ärzte.

[CR46] Staub K, Jüni P, Urner M, Matthes KL, Leuch C, Gemperle G, Bender N, Fabrikant SI, Puhan M, Rühli F, Gruebner O, Floris J (2021). Public health interventions, epidemic growth, and regional variation of the 1918 influenza pandemic outbreak in a Swiss canton and its greater regions. Annals of Internal Medicine.

[CR47] Steere-Williams J (2020). The filth disease. Typhoid fever and the practices of epidemiology in Victorian England.

[CR48] Taddei E (2010). Franz von Ottenthal (1818–1899). Arzt und Tiroler Landtagsabgeordneter.

[CR49] Thiessen M, Thiessen M (2014). Seuchen im langen 20. Jahrhundert. Infiziertes Europa. Seuchen im langen 20. Jahrhundert.

[CR50] Universität Zürich 2022. Emil Rahm. *Matrikeledition der Universität Zürich*. URL: https://www.matrikel.uzh.ch/active/static/17180.htm (1.2.2023).

[CR51] Vagneron, Frédéric 2021. La grippe existe-t-elle? L’énigme de la variabilité d’une maladie à l’heure de la culture bactériologique (1890–1914). *Revue l’anthropologie des connaissances *15 (3).

[CR52] Von Oertzen C, Bittel C, Leong E, van Oertzen C (2019). Keeping prussia’s house in order. Census cards, housewifery, and the state’s data compilation. Working with paper: gendered practices in the history of knowledge.

[CR53] Vasold M (2008). Grippe, Pest und Cholera: eine Geschichte der Seuchen in Europa.

[CR54] Vögele J, Thiessen M (2014). Vom epidemiologischen Übergang zur emotionalen Epidemiologie. Infiziertes Europa. Seuchen im langen 20. Jahrhundert.

[CR55] Vögele J, Knöll S, Noack T (2016). Epidemien und Pandemien in historischer Perspektive.

[CR56] Winkle S (2005). Geißeln der Menschheit: Kulturgeschichte der Seuchen.

[CR57] Witte W (2006). Erklärungsnotstand: die Grippe-Epidemie 1918–1920 in Deutschland unter besonderer Berücksichtigung Badens.

[CR58] Witzler B (1995). Großstadt und Hygiene. Kommunale Gesundheitspolitik in der Epoche der Urbanisierung.

[CR59] Wolff E, Dinges M (1996). Medizinkritik der Impfgegner im Spannungsfeld zwischen Lebenswelt und Wissenschaftsorientierung. Medizinkritische Bewegungen im Deutschen Reich (ca. 1870–ca. 1933).

[CR60] Worboys M (2006). Was there a Bacteriological Revolution in late nineteenth-century medicine?. Stud. Hist. Phil. Biol. & Biomed. Sci.

[CR61] Yersin S (2022). La santé publique entre peste bovine et choléra: l’émergence des institutions fédérales de contrôle des maladies infectieuses en Suisse (1863–1872). Schweizerische Zeitschrift für Geschichte.

